# Bridging computational and clinical strategies for presurgical identification of epileptogenic networks

**DOI:** 10.1002/epi4.70311

**Published:** 2026-07-24

**Authors:** Tena Dubcek, Debora Ledergerber, Kristina Koenig, Adham Elshahabi, Rafael Polania, Lukas Imbach

**Affiliations:** ^1^ Department of Health Sciences and Technology ETH Zurich Zurich Switzerland; ^2^ Swiss Epilepsy Center, Clinic Lengg Zurich Switzerland; ^3^ Neuroscience Center Zurich University of Zurich Zurich Switzerland; ^4^ ETH Zurich Zurich Switzerland

**Keywords:** dynamic network models, epileptic network, neural fragility, seizure onset zone, stereotactic EEG, stimulation mapping

## Abstract

**Objective:**

About one third of epilepsy patients are drug‐resistant. Resective epilepsy surgery remains a key treatment option but depends critically on accurate identification of the seizure onset zone (SOZ), which is still guided mainly by subjective visual inspection of electrophysiological signals. Network‐based metrics derived from intracranial EEG have recently shown promise for SOZ identification, but their evaluation and interpretation have remained disconnected from standard clinical procedures and reasoning.

**Methods:**

We analyzed stereotactic EEG (sEEG) recordings from 20 patients undergoing presurgical evaluation in the interictal state and during clinical mapping via electrical stimulation. We constructed patient‐specific time‐varying dynamic network models and addressed key questions for clinical translation: how sensitive network vulnerability estimates are to the choice among related published metrics, how they depend on the time of the day, and how the resulting conclusions relate to stimulation‐evoked epileptiform discharges as a routine clinical reference for network vulnerability in 5 patients who underwent 50 Hz stimulation mapping. We then simulated virtual thermocoagulation in 6 patients who later underwent thermocoagulation by removing the clinically coagulated nodes and testing whether the resulting network changes went beyond pure network size reduction.

**Results:**

The network metrics correlated with epileptiform discharges evoked by 50 Hz intracranial stimulation in four of five stimulated patients, supporting a link between model‐based network fragility and interictal epileptiform discharges evoked in clinical stimulation mapping. Using virtual thermocoagulation, we quantified the expected network‐level change under model node removal, capturing both local and global effects depending on individual network architecture. Across patients, more fragile network metrics pointed toward clinically defined SOZ contacts and yielded stable conclusions across time, conditions, and perturbation properties, supporting their reliability.

**Significance:**

Together, these findings provide a clinically interpretable calibration of published network vulnerability metrics against routine clinical references, using interictal sEEG data only.

**Plain Language Summary:**

Some people with epilepsy need brain recordings to find the tissue where seizures start. We tested whether computational models built from seizure‐free sEEG recordings can identify vulnerable parts of the epileptic network. The model‐based measures pointed toward clinically defined seizure onset regions, were stable across recording times, and often agreed with responses seen during clinical brain stimulation. These results suggest that interictal network modeling may complement standard presurgical evaluation, although larger prospective studies are needed.

**Key points**

- Interictal sEEG network metrics showed group‐level agreement with clinically defined SOZ contacts.
- Fragility and source–sink metrics were highly correlated and highlighted similar vulnerable contacts.
- Network vulnerability profiles were stable across time of day and perturbation frequency.
- In 4 of 5 stimulated patients, model metrics related to clinical 50 Hz stimulation responses.
- Virtual thermocoagulation estimated local and network‐level effects of clinically targeted nodes.

## 1 INTRODUCTION

Epilepsy is a common neurological disorder, affecting approximately 1% of the global population.^1^ About one third of epilepsy patients are drug‐resistant, meaning they do not respond to two or more anticonvulsive medications.^1^ Alternative therapeutic approaches have been developed, including resective epilepsy surgery^2^, ^3^, ^4^ and brain stimulation.^5^, ^6^ These therapies heavily depend on understanding the epileptic network—a distributed set of brain regions whose abnormal and coordinated activity underlies seizure generation and propagation.^7^, ^8^ A critical step for successful therapy is accurately identifying the resection or stimulation targets that can effectively modulate the pathological network state and achieve seizure freedom.^9^ In clinical practice, this is commonly guided by visual inspection of intracranial EEG (iEEG) to define the seizure onset zone (SOZ),^10^ defined as the region where seizures are first observed.^9^ In addition, interictal epileptiform discharges,^7^, ^11^, ^12^ high‐frequency oscillations^13^, ^14^, ^15^, ^16^, ^17^, ^18^ (HFOs), and other EEG focal or association biomarkers^19^, ^20^, ^21^, ^22^, ^23^, ^24^, ^25^ are used to support localization. However, all these biomarkers represent local or quasi‐static properties of the signal, while it is increasingly recognized that epilepsy is a network disorder involving dynamical interactions across multiple brain regions.^7^ Ideally, clinicians would have access to objective and personalized metrics and models capable of capturing these network dynamics and identifying the network regions most responsible for seizure generation and propagation. Several quantitative and modeling approaches have recently emerged to address this need.^20^, ^26^, ^27^, ^28^, ^29^, ^30^, ^31^, ^32^, ^33^, ^34^, ^35^ Nevertheless, a common challenge is their interpretability within existing clinical frameworks including electrical stimulation‐based mapping,^36^ as well as their feasibility in clinical practice, both necessary for clinician acceptance and practical use in epileptology.

An outstanding example illustrating this issue is the recently introduced metric of neural fragility,^8^, ^37^ a model‐based quantitative index which was retrospectively demonstrated to hold significant potential for guiding surgical planning in drug‐resistant epilepsy. Neural fragility involves the construction of personalized iEEG‐based dynamical models of the (ictal) epileptic network,^8^, ^38^ providing model‐based insights into how easily perturbations of each network node can destabilize the network and trigger the seizure onset.^8^ Despite its promise, neural fragility has not yet seen broad clinical adoption due to both practical and conceptual challenges. The method relies on advanced principles from nonlinear dynamics, which may hinder accessibility and integration into clinical workflows. While it shows strong potential for identifying the SOZ,^8^ its interpretation and alignment with established clinical practices^36^, ^39^, ^40^, ^41^ remain active areas of investigation. Other concerns regarding the generalizability and robustness of neural fragility include its sensitivity to different types of perturbations, the source data selection (particularly whether widely available interictal recordings are already sufficient for reliable conclusions), and the potential inclusion of subcortical structures. Moreover, other proposed metrics sharing similar rationales^42^ have not been adequately differentiated or discussed comparatively, while their reported performance often hinges on the choice of machine learning classifiers and summary scores.^43^

In this study, we focused on clinical translation by providing a calibration of network vulnerability metrics against routine clinical references using exclusively interictal recordings from stereotactic deep electrodes (sEEG). Based on sEEG recordings of 20 epilepsy patients, we constructed dynamic network models^8^, ^38^ and evaluated the earlier promising network‐based metrics for each channel: outgoing fragility, incoming fragility, source influence, and sink connectivity.^8^, ^41^, ^42^ We compared and dynamically interpreted these metrics against each other because several practical choices are not obvious for clinical adoption: which metric to use, how many metrics are needed to obtain a reliable input for presurgical evaluation, and which data are sufficient for robust estimation. We found high agreement across metrics and stable conclusions across time and perturbation settings, suggesting that similar information can be obtained with lower computational burden while supporting the robustness of the resulting vulnerability readouts for clinical use. Motivated by the fact that presurgical evaluation of epilepsy often involves sEEG‐based brain stimulation mapping,^36^ which essentially serves as an experimental counterpart to neural fragility, we analyzed how the level of epileptiform discharges evoked by 50 Hz intracranial stimulation relates to the network‐based metrics. In addition, we used virtual thermocoagulation to estimate network‐level consequences of clinically motivated interventions. While related virtual cortical resection approaches have studied node‐by‐node removal in functional connectivity networks,^44^ here we focus on clinically executed multi‐node thermocoagulation sets and benchmark their network‐level effects within time‐varying dynamical interaction models. This could provide a clinically anchored way to explore whether locally targeted interventions may have broader effects on the remaining large‐scale epileptic network.

## 2 METHODS

### 2.1 Patient population and electrode implantation

**FIGURE 1:**
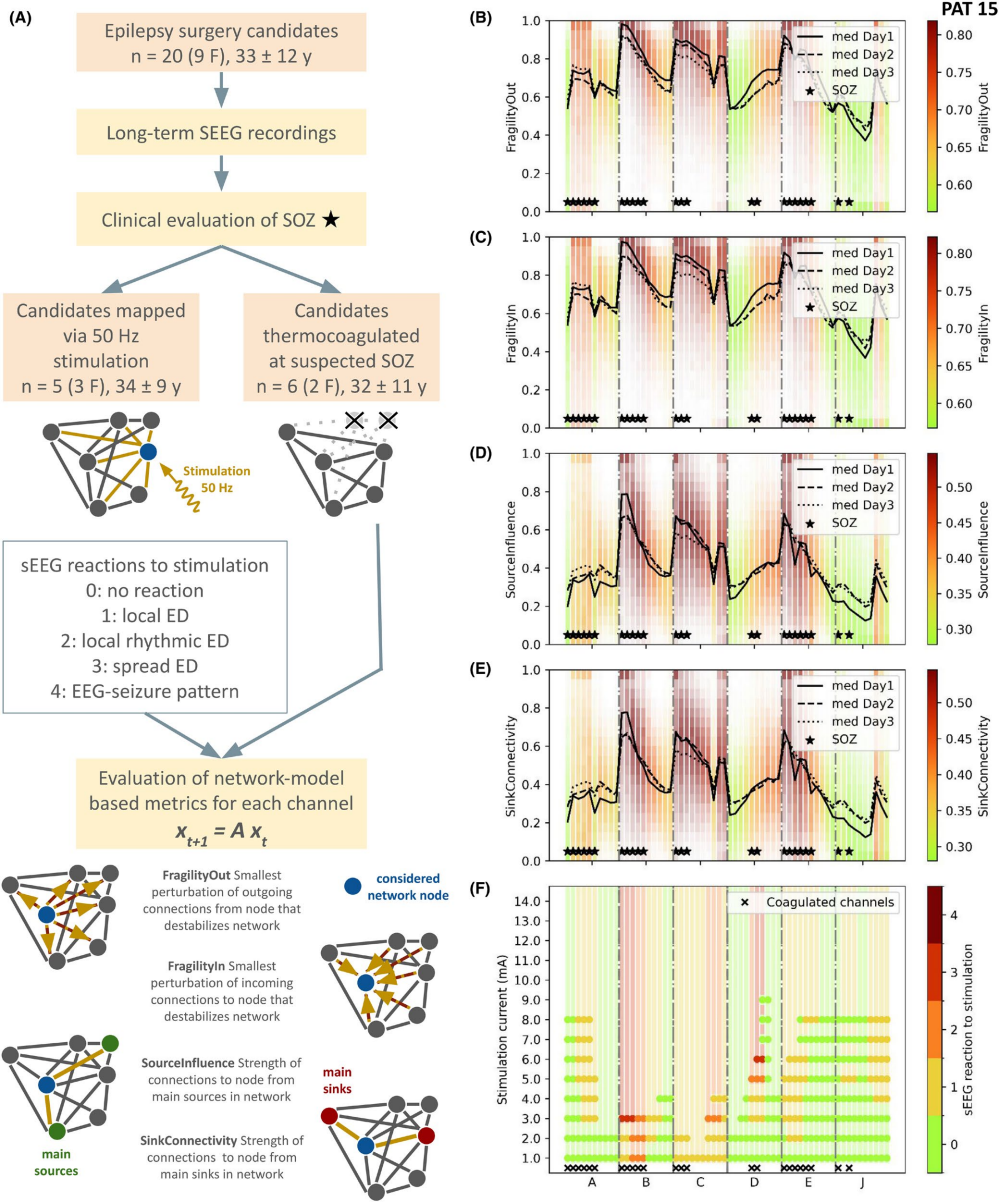
Overview. (A) Schematic of computational and clinical pipeline. (B–E) Dynamic network‐based metrics for one exemplary patient. Black lines indicate daily medians. Shaded distributions show channel‐wise values per day, colors reflect daily medians for visual comparison. Black stars mark clinically defined SOZ channels. Electrode names are shown at the bottom; each electrode contains multiple contacts. (B) Outgoing fragility. (C) Incoming fragility. (D) Source influence. (E) Sink connectivity. (F) Quantified reactions to 50 Hz stimulation in same patient. Filled circles denote tested electrode–current pairs. Background shading denotes maximal observed reaction per electrode across all tested currents. Black crosses mark thermally coagulated channels.

We included 20 patients with pharmacoresistant focal epilepsy (9 females, mean age 33 ± 12 years) who underwent iEEG recordings with stereo electroencephalography (sEEG) as part of their presurgical evaluation at the Swiss Epilepsy Center (Figure 1A). All patients were candidates for epilepsy surgery. The sEEG procedure (including reduction of medication, electrical stimulation, and duration of recording) was performed based solely on clinical decisions.

The implantation strategy was individualized for each patient and based on prior noninvasive evaluations. Electrodes from DIXI‐medical Microdeep® and AD‐TECH‐medical Spencer were stereotactically implanted in cortical and subcortical brain regions suspected to be involved in seizure generation. Electrode locations were verified using preimplantation and postimplantation magnetic resonance imaging (MRI). A detailed overview of implanted electrodes, antiseizure medications, and clinical profiles of all patients is found in Table 1.

**TABLE 1:** Patient demographics: Age range, sex, antiseizure medications during iEEG recording, whether mapping by electrical stimulation was performed, whether thermocoagulation was performed, and whether resection was performed and with which outcome (Engel class).

PAT	Age range	Sex	ASM at iEEG	Electr. Stim.	Therm. Coag.	Resect.	Resect. Outcome
1	26–30	M	LEV, LCM, CLB, PER	N	N	N	–
2	26–30	F	BRV, CLN	N	N	N	–
3	26–30	F	LEV, PER	N	N	Y	IA
4	56–60	M	BRV, LCM	N	N	N	–
5	26–30	M	LEV, LTG, PER	N	N	Y	IA
6	26–30	M	LTG, BRV	N	N	N	–
7	31–35	M	ZNS	N	N	Y	IIB
8	16–20	M	BRV, LTG	N	N	Y	IA
9	16–20	M	LEV, LCM	N	N	N	–
10	06–10	F	OXC	Y	N	Y	III
11	16–20	M	LEV, OXC	Y	Y	Y	IA
12	41–45	M	LTG, VPA, LCM	Y	Y	N	–
13	11–15	F	PER, OXC	N	N	Y	IB
14	36–40	F	CEN	N	N	N	–
15	21–25	F	PHE, BRV, ESL, ZNS, CLB	Y	Y	Y	III
16	31–35	F	PER, LEV, LTG	N	N	N	–
17	41–45	M	ESL, PER	Y	Y	N	–
18	26–30	M	LTG	Y	Y	N	–
19	31–35	F	BRV, CBD, LCM	Y	Y	N	–
20	41–40	F	LEV, PHT, PGB, CLZ	Y	N	N	–

Abbreviations: BRV, Brivaracetam; CBD, Cannabidiol; CEN, Cenobamate; CLB, Clobazam; CLN, Clonazepam; CLZ, Clonazepam; ESL, Eslicarbazepine acetate; LCM, Lacosamide; LEV, Levetiracetam; LTG, Lamotrigine; OXC, Oxcarbazepine; PER, Perampanel; PGB, Pregabalin; PHE, Phenytoin; PHT, Phenytoin; VPA, Valproate; ZNS, Zonisamide.

The study was approved by the local ethical committee (Kantonale Ethikkommission Zürich, Approval PB 2016–02055), and all patients or their legal representatives provided written informed consent in accordance with the Declaration of Helsinki. However, this consent did not include a provision for making individual data freely accessible.

### 2.2 Clinical mapping and radiofrequency thermocoagulation

A subgroup of five candidates underwent 50 Hz stimulation via electrodes from Micromed to map brain activity and identify functional eloquent brain regions (Figure 1A). The sEEG reactions (changes in baseline EEG) were quantified by two independent clinicians using the following scale: (0) no reaction, (1) local epileptiform discharge, (2) local rhythmic epileptiform discharges, (3) spread epileptiform discharges, (4) EEG‐seizure pattern. We note that stimulation was performed according to clinical indication and was not necessarily applied to all contacts. Contacts were selected by the clinical team based on the suspected seizure network, unclear epileptogenic or functional relevance from passive recordings, and the need to map eloquent cortex. The patient‐wise plots in Supplementary materials show all recorded contacts and indicate which contacts were stimulated and with which response.

During the presurgical evaluation of all candidates, the responsible clinicians defined the suspected SOZ based on the combined analysis of sEEG and scalp EEG during the recorded seizures. Based on this evaluation, each sEEG channel was classified as either SOZ or non‐SOZ and treated accordingly in all subsequent analyses. These labels represent the retrospective clinical SOZ assessment used for patient management and should not be treated as an independent ground truth. A subgroup of 6 candidates underwent local brain lesioning by radiofrequency thermocoagulation (coagulation device from Inomed Medizintechnik GmbH) around electrode contacts that were identified as belonging to SOZ, but not part of an eloquent area^45^ (Figure 1A).

### 2.3 Data preprocessing

All recorded sEEG signals were re‐referenced using common average referencing, notch‐filtered at 50 Hz to remove line‐noise contamination, low‐pass filtered at 100 Hz, and resampled to $f_{s}$ = 200 Hz. Resampling to a lower sampling frequency was motivated by computational efficiency in estimating the best‐fit linear models, as frequencies higher than 100 Hz were not considered relevant for the present analysis. No segments containing seizures were included. All recordings were visually inspected to ensure that no major non‐neurophysiological artifacts were included (in contrast to scalp EEG, sEEG recordings are generally very stable and contain few visually noticeable artifacts). Interictal epileptiform discharges were not removed, because they represent clinically relevant interictal network activity and were part of the signal state analyzed by the model.

### 2.4 Dynamic network models of sEEG

We modeled the preprocessed interictal sEEG dynamics by a discrete‐time linear approximation of the form

$$x\left(t+1\right)=A\left(t\right)\;x\left(t\right),$$

where $x\left(t\right)\in R^{n}$ is the multichannel sEEG signal at time *t*, and $A\in R^{n\times n}$ is the state transition matrix capturing effective directed interactions between channels. This linear model should not be interpreted as a complete model of nonlinear seizure generation mechanisms. Rather, it is a local, finite‐dimensional approximation of short‐window sEEG dynamics, motivated by Koopman operator‐based views of nonlinear systems, where nonlinear dynamics can be approximated through linear evolution of observed signal coordinates over short time intervals.^46^, ^47^ In this framework, the fitted matrix *A* summarizes one‐sample‐ahead effective interactions between recorded channels and provides a tractable network model for stability‐related metrics. This model class is also consistent with the original fragility and source–sink frameworks,^8^, ^41^, ^42^ where similar one‐step linear dynamical models showed clinical value for SOZ localization and outcome prediction. The matrix *A* was estimated independently over sliding windows of 0.5 s duration and with 0.5 s shift. In each window, A was obtained by solving a regression model with l2‐regularization $\alpha_{\text{Ridge}}=10^{-4}),$ yielding a time‐varying sequence of linear models that formed the basis of all subsequent metrics. Ridge regularization was used to stabilize the short‐window least‐squares estimation and reduce sensitivity to noise and channel collinearity. All metrics in this work refer to properties of the estimated matrices *A*.

Multiple 10‐min long sEEG recordings were analyzed for each patient. In contrast to previous studies focused on ictal activity, our analysis was conducted entirely on interictal recordings, which offer more stable conditions, better comparability across patients, and avoid the confounding effects of seizure‐related dynamics. For all patients, data recorded in the morning, afternoon, and early night two days after implantation were used for analyzing temporal robustness of the network‐based metrics. For patients who underwent stimulation mapping, data recorded at approximately the same time in the three consecutive days prior to stimulation were used. For patients who underwent radiofrequency thermocoagulation, the mean of morning, afternoon, and night metric values was used in model‐based virtual thermocoagulation.

### 2.5 Outgoing and incoming fragility

To quantify the susceptibility of each node to destabilizing the network, we computed neural fragility based on structured perturbation analysis.^8^ Intuitively, fragility reflects how close a given brain region is to tipping the whole network into a seizure‐prone state: fragile nodes are those where even small changes in their connections can destabilize the system, making them likely contributors to seizure initiation.

For each estimated matrix $A\left(t\right)$, frequency dependent inverse fragility is defined as the minimal norm of a perturbation to the kth row or column of A that pushes one of its eigenvalues λ to the unit circle,

$$\text{invfra}g_{k}\left(\omega ,t\right)=\min_{\Delta_{k}}\left|\Delta_{k}\right|\;s.t.\left(A\left(t\right)+\Delta_{k}\right)v=e^{\mathrm{i\omega}}v.$$

Row perturbations (${∆}_{k}=e_{k}\Gamma^{T}$) simulate changes to incoming connections to node *k*, while column perturbations $({∆}_{k}=\Gamma e_{k}^{T}$ affect outgoing projections from node *k*. The constrained minimization can be rewritten as

$$\text{invfra}g_{k}\left(\omega ,t\right)=\min_{\Gamma }\left|\Gamma \right|\;s.t.B_{k}\left(\omega ,t\right)\Gamma =b,$$

with, for row perturbations,

$$a_{k}\left(\omega \right)={\left(A-e^{\mathrm{i\omega}}I\right)}^{-1}e_{k}\equiv a_{k,r}+ia_{k,i}\in C^{n\times 1},$$

$$B_{k}\left(\omega ,t\right)=\left(\begin{array}{c} a_{k,i}^{T}\\ a_{k,r}^{T} \end{array}\right)\in R^{2\times n},\;b=\left(\begin{array}{c} 0\\ -1 \end{array}\right)\in R^{2\times 1},$$

and for column perturbations,

$$a_{k}\left(\omega \right)=e_{k}^{T}{\left(A-e^{\mathrm{i\omega}}I\right)}^{-1}\equiv a_{k,r}+ia_{k,i}\in C^{1\times n},$$

$$B_{k}\left(\omega \right)=\left(\begin{array}{c} a_{k,i}\\ a_{k,r} \end{array}\right)\in R^{2\times n},\;b=\left(\begin{array}{c} 0\\ -1 \end{array}\right)\in R^{2\times 1}.$$

The closed‐form solution, obtained using the Lagrange multipliers method, is

$$\Gamma \left(\omega ,t,k\right)=B_{k}^{T}{\left(B_{k}B_{k}^{T}\right)}^{-1}b.$$

Node fragility is then obtained from

$$\text{invfra}g_{k}\left(t\right)=\min_{\omega }\left|\Gamma \left(\omega ,t,k\right)\right|,$$

with $\omega =2\mathrm{\pi f}/f_{s}$, $f\in \left[0.5,100\right]$ Hz, by inversely linearly normalizing $\text{invfra}g_{k}$ across all nodes k within each time window t,

$$\mathrm{Fra}{\text{gility}}_{k}\left(t\right)=\frac{\max_{j}\text{invfra}g_{j}\left(t\right)-\text{invfra}g_{k}\left(t\right)}{\max_{j}\text{invfra}g_{j}\left(t\right)-\min_{j}\text{invfra}g_{j}\left(t\right)}.$$

We computed normalized fragility separately for column (outgoing) and row (incoming) perturbations, resulting in two complementary fragility maps per time window, $\mathrm{Fra}{\text{gility}}_{k}^{C}\left(t\right)$ and $\mathrm{Fra}{\text{gility}}_{k}^{R}\left(t\right)$. Since outgoing and incoming fragility proved extremely highly correlated (see Results), we also defined a single fragility metric $\text{Fragilit}y_{k}\left(t\right)$ as their average. To obtain time‐aggregated fragility profiles, the normalized fragility values were summarized across windows by taking the median over time.

### 2.6 Source influence and sink connectivity

We also extracted source influence and sink connectivity metrics^42^ from the dynamic network models. Intuitively, the source–sink framework characterizes the directional flow of influence in the network: sources are nodes that strongly drive others, while sinks are strongly driven. In epilepsy, seizure onset zones seem often to appear as abnormal sinks, reflecting their heightened recruitment by surrounding regions.^42^

Based on the estimated matrix e computed the total absolute incoming and outgoing weights for each node *k*

$$\begin{array}{c} r_{k}=\sum_{j=1}^{N}|A_{\mathrm{kj}}|,c_{k}=\sum_{j=1}^{N}|A_{\mathrm{jk}}|, \end{array}$$

and their rank‐normalized values $rr_{k}$ and $cr_{k}$ across all nodes. The sink index was the proximity to the ideal sink point $\left(1,\frac{1}{N}\right)$ in rank space

$$\sin k_{k}=\sqrt{2}-\left|\left(rr_{k},cr_{k}\right)-\left(1,\frac{1}{N}\right)\right|,$$

and the source index analogously to $\left(\frac{1}{N},1\right)$. These indices were then used to compute higher‐order connectivity features, source influence,

$$\inf l_{k}\left(t\right)=\sum_{j=1}^{N}\left|A_{\mathrm{kj}}\left(t\right)\right|\;\text{sour}{\mathrm{ce}}_{j}\left(t\right),$$

and sink connectivity,

$$\mathrm{con}n_{k}\left(t\right)=\sum_{j=1}^{N}\left|A_{\mathrm{kj}}\left(t\right)\right|\;\sin k_{j}\left(t\right),$$

and their normalized versions

$$\text{SourceInfluenc}e_{k}\left(t\right)=\frac{\max_{j}\;\inf l_{j}\left(t\right)-\inf l_{k}\left(t\right)}{\max_{j}\inf l_{j}\left(t\right)-\min_{j}\inf l_{j}\left(t\right)},$$

$$\text{SinkConnectivit}y_{k}\left(t\right)\frac{\max_{j}\;\mathrm{con}n_{j}\left(t\right)-\mathrm{con}n_{k}\left(t\right)}{\max_{j}\mathrm{con}n_{j}\left(t\right)-\min_{j}\mathrm{con}n_{j}\left(t\right)}.$$

Since these two measures later proved to be highly correlated (see Results), we also defined a single metric as their average,

$$\begin{array}{c} \text{SourceSin}k_{k}\left(t\right)=\frac{1}{2}\left(\text{SourceInfluenc}e_{k}\left(t\right)+{\text{SinkConnectivity}}_{k}\left(t\right)\right). \end{array}$$

Time aggregation was done analogously as for fragility.

### 2.7 Statistical methods

Clinical data is rarely Gaussian due to relatively small sample sizes and inter‐patient variability. We therefore used nonparametric, permutation‐based analyses for all statistical comparisons. Permutation tests were used to assess correlations between network metrics by permuting channel‐wise metric values within each patient. For group comparisons, such as between SOZ and non‐SOZ channels, between reacting and non‐reacting channels, or between different times of day, group labels were permuted while preserving the underlying metric values. In all cases, the observed statistic was compared to the empirical null distribution generated from 10 000 permutations.

For visualization, statistical significance is denoted by asterisks: *(*p* < 0.05), **(*p* < 0.01), and ***(*p* < 0.001). The significance threshold was set at α=0.05, and Bonferroni correction was applied when performing multiple comparisons within individual patients (e.g., for pairwise metric correlations or daytime condition effects).

In analyses involving thermocoagulation, we computed a null distribution of network metric changes using repeated random removal of channels. The null distributions obtained for each channel were then bootstrapped with 5000 repetitions, and their 97.5th percentile was used to estimate whether changes after removal of clinically coagulated nodes exceeded the chance and effects that come exclusively from a change in network size. This empirical approach provided a patient‐specific control for interpreting network effects.

## 3 RESULTS

The sEEG recordings of each patient were used for data‐driven extraction of the network dynamics (encoded in the dynamic connectivity matrices A) and the evaluation of the corresponding network‐based metrics for each sEEG channel (Figure 1A). Outgoing and incoming fragility was defined as the normalized time‐median of the smallest perturbation to outgoing and incoming connections of a node that destabilizes the whole network (Figure 1B,C). Source influence reflected the normalized time‐median of the strengths of connections from the main sources in the network (Figure 1D), while sink connectivity reflected the normalized time‐median of the strengths of connections from the main sinks in the network (Figure 1E). These network‐derived measures were then compared with the severity of epileptiform discharges evoked during clinical 50 Hz intracranial stimulation mapping (Figure 1F). The detailed results for all other patients can be found as Supplementary materials.

### 3.1 Network‐based metrics point toward SOZ and remain stable despite EEG variability

**FIGURE 2:**
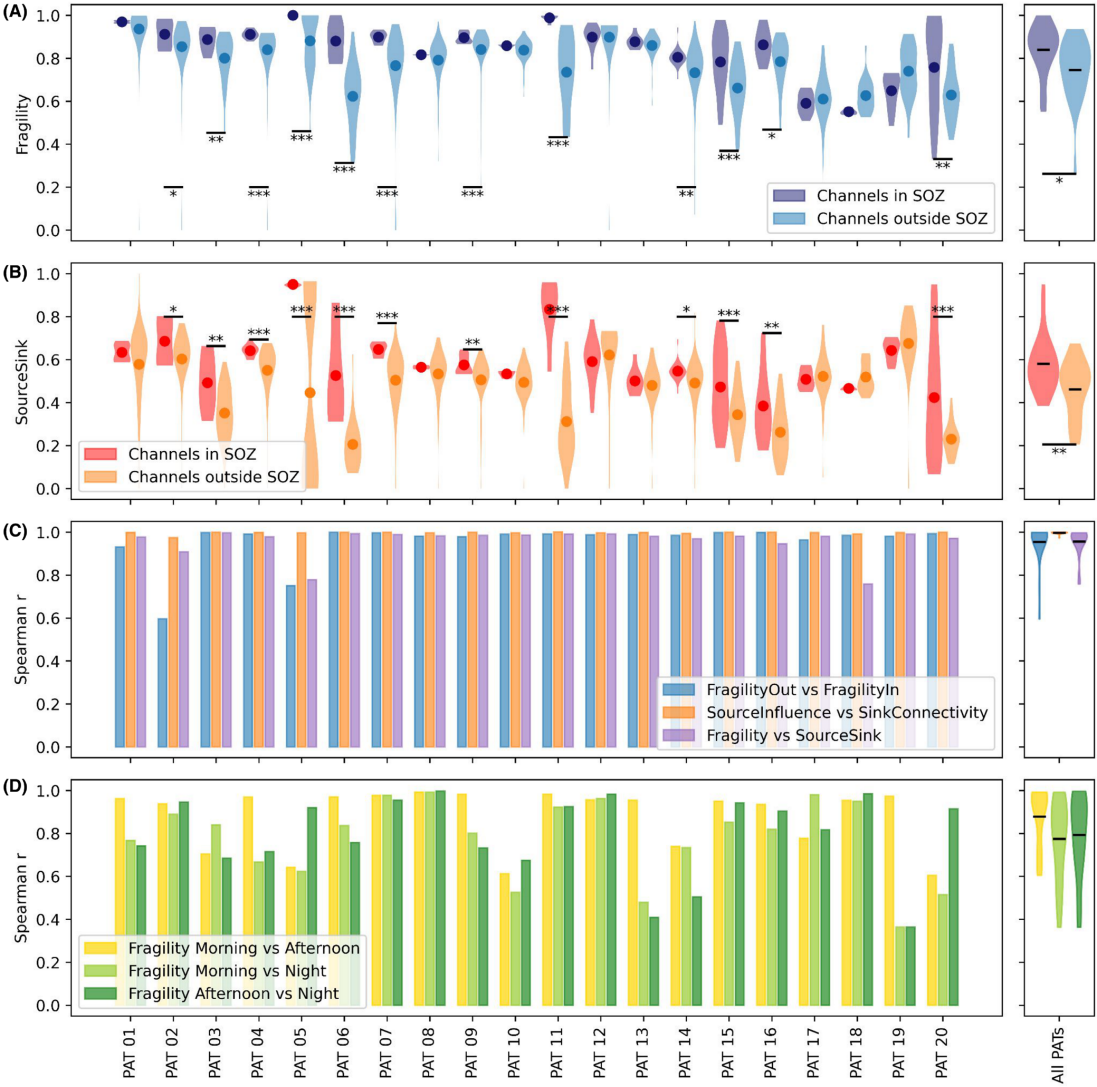
Network‐based metrics point toward the SOZ and are extremely robust. (A) Left: Patient‐wise comparison of fragility distributions (average of outgoing and incoming fragility) between channels inside and outside the SOZ. Stars indicate levels of statistically significant differences in means based on permutation testing. Right: Distribution of patient‐level means for channels inside and outside the SOZ. Black horizontal lines denote the population means. (B) Same as A, but for the source–sink metric (average of source influence and sink connectivity). (C) Left: Patient‐wise Spearman correlations between outgoing and incoming fragility, source influence and sink connectivity, and the means of fragility and source–sink metrics. Right: Population distributions and means of the correlation values. (D) Left: Spearman correlations of fragility metrics computed from recordings at different times of day. Right: Population distributions and means of the correlation values.

We first assessed, using exclusively interictal data from patients with both cortical and subcortical electrode implantations, the ability of these network‐based metrics to point toward the clinically defined SOZ. In a personalized manner, we compared the distributions of time‐aggregated fragility and source–sink values (see Methods for definitions of Fragility and SourceSink) between EEG channels inside and outside the SOZ for each patient (Figure 2A,B left). The metrics evaluated from sEEG data recorded in the morning showed significant differences in distribution means inside and outside the SOZ for 12 of the 20 patients. Interestingly, patients who showed significant differences in the fragility distribution also showed significant differences in the source–sink distribution, although the significance level could vary, already suggesting that these network‐based metrics provide consistent vulnerability readouts and tend to highlight similar regions as clinically defined SOZ candidates. By comparing the distributions of patient‐wise means inside and outside the clinically defined SOZ (Figure 2A,B right), we could show that fragility and source–sink values are significantly higher in SOZ than in non‐SOZ regions on a group level: fragility ${∆}_{F}=0.09,p_{F}=0.017$, and source–sink ${∆}_{\mathrm{SS}}=0.12,p_{\mathrm{SS}}=0.003$.

Next, we aimed to clarify the robustness of the conclusions derived from different network‐based metrics. We analyzed the differences in channel dependences of outgoing, incoming fragility, source influence and sink connectivity for each patient. We observed statistically significant correlations for all patients and all metric type pairs (Figure 2C left). On the population level, the Spearman correlations showed the following confidence intervals: outgoing versus incoming fragility $r\in \left[0.92,0.99\right]$, source influence versus sink connectivity *r* ∈ [0.96,1.00], and fragility versus source–sink $r\in \left[0.70,0.88\right]$ (Figure 2C right). Thus, despite their different definitions, all four metrics converge on highlighting the inter‐regional dynamics and regions driving network instability in a consistent manner relevant for clinical translation.

Finally, we assessed sensitivity to the choice of data time span by comparing results derived from morning, afternoon, and night sEEG recordings (Figure 2D left). We found that the distributions of channel fragility remained significantly correlated across time for all patients. On the population level, the Spearman correlations showed the following confidence intervals: morning versus afternoon $r\in \left[0.79,0.97\right]$, morning versus night $r\in \left[0.68,0.86\right]$, and afternoon versus night $r\in \left[0.70,0.88\right]$ (Figure 2D right). We further showed that the choice of perturbation frequency used to quantify node fragility had only a weak and smooth impact on normalized fragility values and the relative ordering of channels was preserved across frequencies in the range 0.5–100 Hz (see Supplementary materials). Together, these findings indicated that the identified vulnerable regions remain highly stable across time, conditions, and frequency choices, informing which aspects of data selection are less critical for reliable estimation in a clinical setting.

### 3.2 Model‐based fragility correlates with sEEG reactions to stimulation

We used electrical stimulation at 50 Hz in a subset of 5 patients during presurgical evaluations to map eloquent brain areas and identify the SOZ. EEG reactions to stimulation quantify how the latter affects the departure from the steady state of individual brain dynamics. This approach offers a way to experimentally probe the network's reactivity, similar in spirit to model‐based perturbation analysis, where simulated perturbations are used to assess how close a given network node is to destabilizing the system. Both approaches aim to reveal the parts of the network that are most capable of driving pathological activity. We therefore expected channels that respond strongly to stimulation to also show high fragility or influence in the model‐based metrics.

**FIGURE 3:**
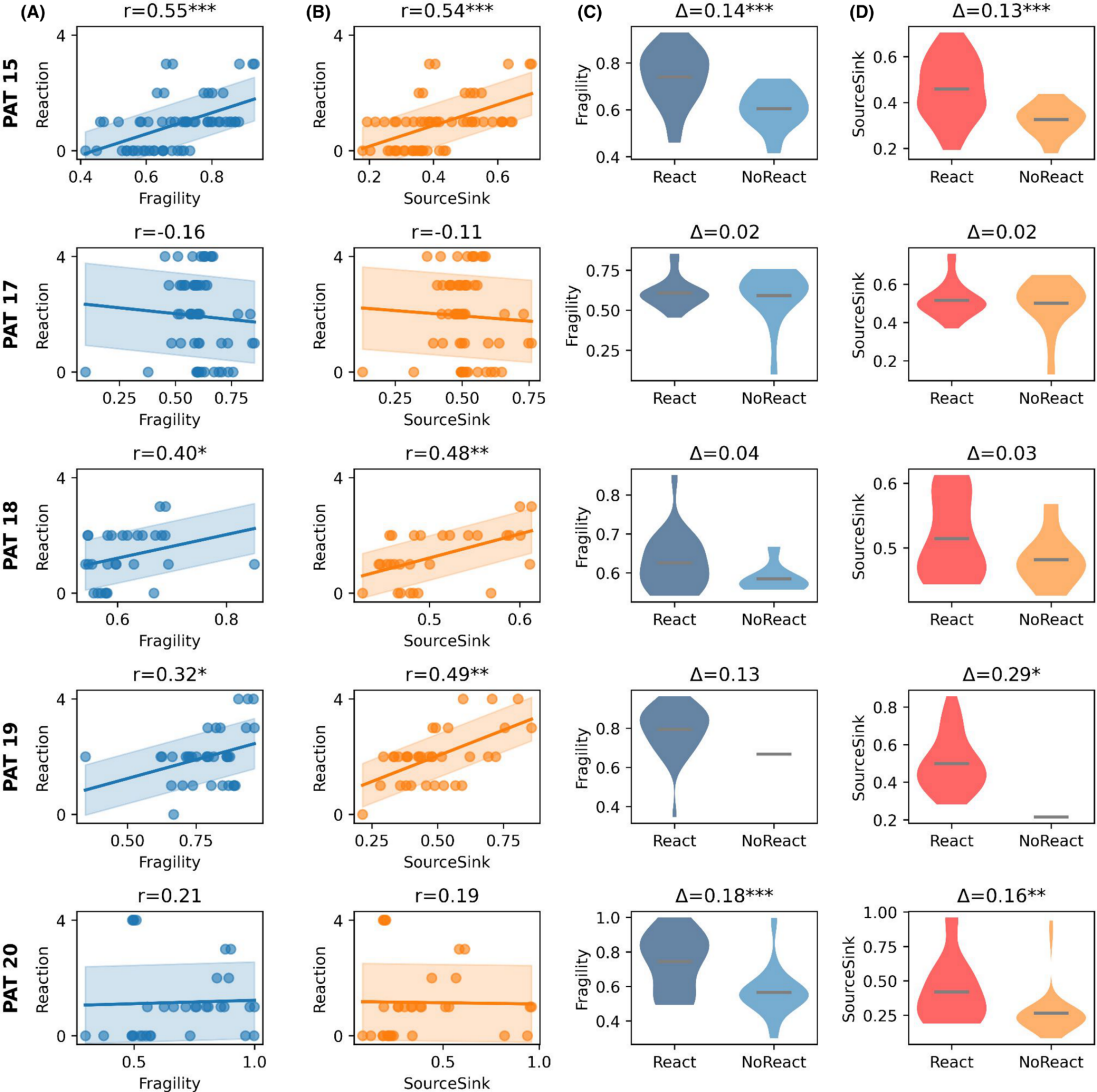
Model‐based fragility and source–sink metrics correlate with stimulation‐evoked sEEG responses in individual patients. (A) Correlation of model‐based fragility values and level of sEEG reaction to 50 Hz direct electrical stimulation, used to map eloquent brain areas and identify SOZ in presurgical evaluation. The Spearman correlation values and statistical significance based on permutation testing are shown at the top of each subplot. (B) Same as A, but for source–sink metric. (C) Distributions and means (vertical lines) of model‐based fragility values in sEEG channels that reacted to stimulation and those that did not. The difference in means and the corresponding statistical significance based on permutation testing are shown at the top of each subplot. (D) Same as C, but for source–sink metric.

For each patient, we correlated the values of the fragility and source–sink metrics with the corresponding stimulation‐assessed reactions, and we compared the metric means between reacting and non‐reacting channels (Figure 3). Four of the five stimulated patients showed positive correlations between model‐based metric values and the strength of stimulation‐evoked responses during presurgical evaluations, with statistical significance varying across patients and metric types (see Figure 3A,B for individual Spearman r values). The difference in mean network metric values between channels that reacted to stimulation and those that did not was positive for all patients, again with statistical significance that varied depending on the patient and metric (see Figure 3C,D for individual ∆ and p values).

### 3.3 Estimating consequences of virtual thermocoagulation via network models

Given that radiofrequency thermocoagulation and surgical interventions target specific brain regions suspected to be responsible for initiating seizures, we asked whether our models and model‐based metrics could help estimate how removing these regions would impact overall network stability. From a modeling perspective, such regions may correspond to nodes whose perturbation induces pathological transitions toward unstable brain states. When performing thermocoagulation or brain resection, the changes in the brain occur locally, but what ultimately matters is how these changes propagate through the broader network. Focusing solely on the SOZ may therefore be shortsighted, as it does not capture downstream or network‐wide consequences that may be critical for seizure generation or control. Network models may help uncover these non‐local effects.

**FIGURE 4:**
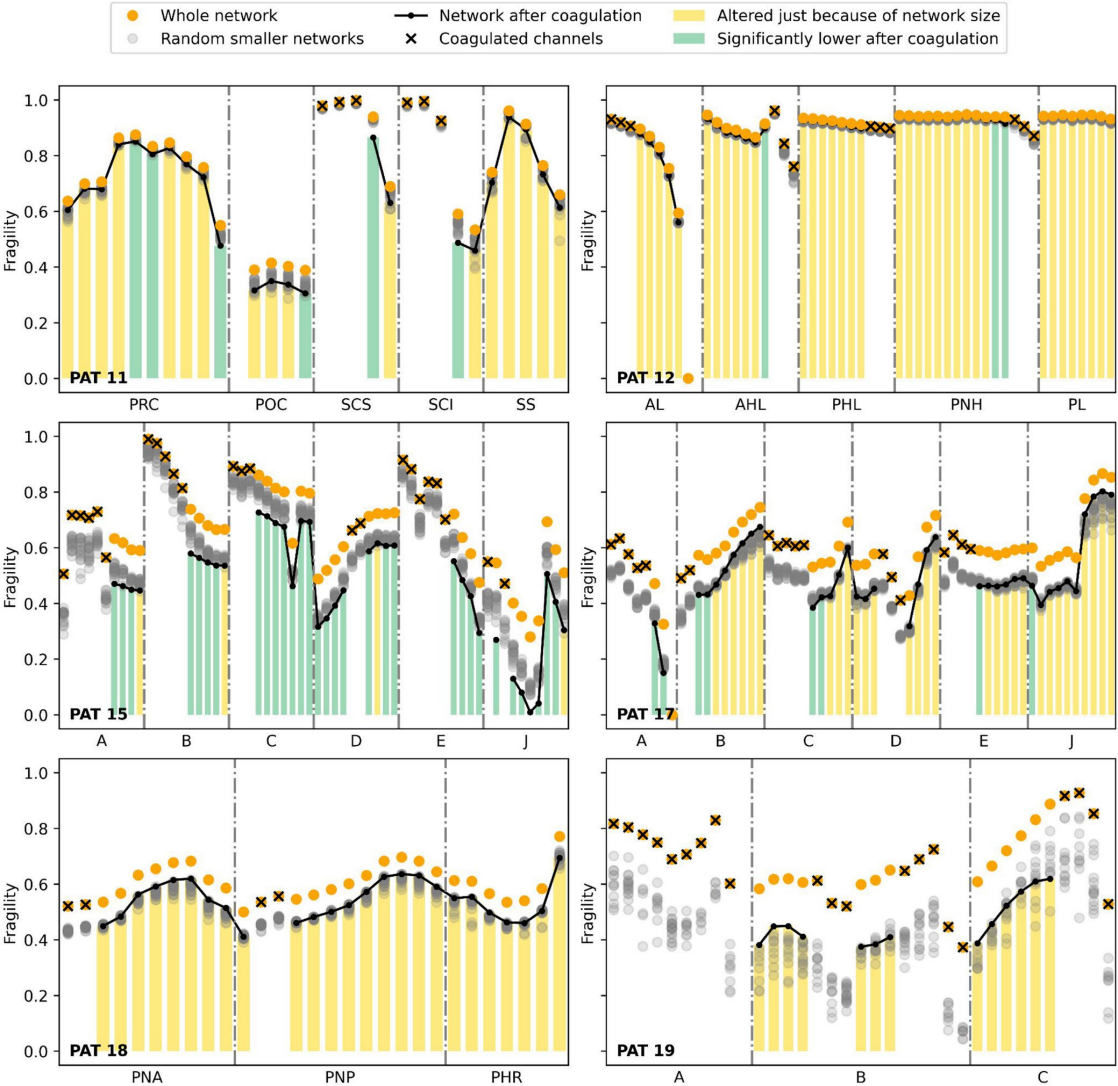
Predicting network‐level consequences of brain thermocoagulation using personalized virtual resection models. Each subplot corresponds to one patient who underwent thermocoagulation of selected electrode contacts. Orange markers denote fragility values for whole, intact network. Black lines denote fragility values after virtual removal of clinically coagulated nodes, signaled with black crosses. Gray transparent markers show the null distribution of fragility values obtained after repeatedly removing the same number of randomly selected nodes. The comparison with the 97.5th percentile of the corresponding bootstrapped distribution is then used to determine the specificity of the observed changes in fragility for the virtual removal of coagulated nodes: Channels with significantly decreased fragility after virtual thermocoagulation are colored in green, the rest in yellow.

To address this, we used personalized network models to post‐hoc simulate the effects of node removal via thermocoagulation. In this simulation, each removed node represents an sEEG contact/local recorded region, and node removal corresponds to deleting this state variable together with all modeled incoming and outgoing effective interactions. For each of the 6 patients who underwent thermocoagulation, we evaluated how fragile the nodes were when the full network was intact, and how this changed after virtual removal of the nodes that had been clinically coagulated (Figure 4). To distinguish changes caused by the removal of nodes driving network instability from those caused merely by a reduction in network size, we computed a null distribution by repeatedly removing the same number of randomly selected nodes. This allowed us to determine, for each patient, the network‐driven consequences of the local changes that were chosen clinically. We observed patients in whom the entire network became significantly more resilient and less fragile after the virtual removal of a few key nodes (mostly green in Figure 4), but also patients in whom the virtual removal of the coagulated nodes had almost no significant consequences for the remaining network. Interestingly, in patients whose network‐driven changes were relatively weak, we could still observe local effects near the coagulated channels (green changes adjacent to coagulated contacts in Figure 4). This can be seen as an internal validation, showing that local effects are expected because of strong nearby connections, while global changes arise only when permitted by the patient's network architecture.

## 4 DISCUSSION

In this study, we systematically evaluated a set of earlier proposed network‐based metrics, derived from interictal sEEG recordings, to characterize the stability and responsiveness of the epileptic brain network in focal drug‐resistant epilepsy. Going beyond traditional biomarker development, we focused on bridging the model‐based metrics with conventional neurophysiological readouts. We demonstrated that these interictal metrics often correlate with sEEG responses evoked by intracranial stimulation and can quantify the network effects of virtual thermocoagulation, thereby offering a personalized, model‐based perspective on stimulation and resection strategies. To support their interpretability and reliability, we first established that the four metrics, although conceptually distinct, are highly correlated, discriminate SOZ from non‐SOZ regions, and remain remarkably stable across times of day and perturbation properties. This coherence suggests that the metrics capture a common, high‐level property of network dynamics, likely linked to the nonlinear functional connectivity and global stability landscape of the epileptic brain,^7^ and that similar clinical insights may be obtained by focusing on the most computationally efficient metric. Although fragility is conceptually close to the clinical concept of seizures as destabilizations of otherwise stable healthy network states, its computation involves a frequency sweep and is therefore computationally more expensive than source–sink metrics, which are computed directly from the estimated network models. The high correlations of different metric values across all patients thus suggest that, in practice, the evaluation of a single computationally efficient and robust metric (source or sink) can be a pragmatic choice of readout for clinical translation.

Importantly, our findings were obtained from interictal recordings alone, avoiding the clinical and technical challenges associated with capturing ictal activity. In contrast to conventional biomarkers like spikes or HFOs,^11^, ^12^, ^13^, ^14^, ^16^ the considered approach offers a network‐based quantitative framework that reduces dependence on visual interpretation and shows stable results across recording times. While we have confirmed that fragility‐related measures are typically high in clinically defined SOZ regions,^8^ they were sometimes high also outside the SOZ, highlighting additional potentially unstable nodes. These might represent previously unrecognized regions of network involvement or even targets that could be used for neuromodulation in multifocal epilepsies.

In the subset of patients who underwent 50 Hz stimulation mapping, we found significant correlations between the stimulation‐evoked changes in epileptiform discharges and the fragility‐based metrics, reinforcing the interpretation of fragility as a proxy for network responsiveness to external perturbation. This comparison should be interpreted in the context of clinical stimulation practice, as stimulation was targeted to selected contacts rather than performed exhaustively across all implanted contacts. Nevertheless, it provides a direct conceptual bridge between the model‐based perturbation analysis and clinical stimulation mapping, as both approaches probe how the epileptic network responds to perturbation. While the clinical scoring of stimulation responses^36^, ^39^, ^40^ is often limited by subjectivity and poor granularity, model‐based network fragility may provide a quantitative and scalable complement to this process. In large‐scale implantations with more than 10 electrodes and up to 100 contacts, exhaustive stimulation becomes time‐wise unfeasible. Thus, these findings motivate future prospective testing of whether model‐derived vulnerability could help prioritize contacts for targeted stimulation.

We hypothesized that the removal of instability‐driving network nodes would lead to significantly higher resilience to disruption (lower fragility) in the remaining network, while removal of noninvolved or misidentified nodes would have negligible impact. To test this, we used model‐based virtual thermocoagulation to evaluate how network fragility changes after virtually removing nodes that had been coagulated based on clinical SOZ identification. This node removal should be understood as an instantaneous and discrete functional disconnection simulation within the effective network model. The node‐based formulation follows the clinical definition of thermocoagulation by targeted contacts/regions, whereas model edges represent inferred effective interactions that cannot be uniquely assigned to specific biological connections. The analysis therefore does not capture spatially graded tissue damage, partial axonal disruption, or later reorganization. Modeling these effects would require additional biophysical assumptions and goes beyond the data‐driven effective network framework used here. Nevertheless, our finding that in some patients the resulting network became significantly more resilient suggests that brain tissue around the thermocoagulated channels had a genuine destabilizing role. In other patients, the network‐level effect was weak or absent, highlighting inter‐patient variability and potential limitations of local interventions. Interestingly, in almost all cases, the most prominent changes were located near the removed nodes, which might reflect the underlying neuroanatomy and, consequently, stronger connections in the network model.

Several limitations should be acknowledged. First, the overall cohort size was modest, and the stimulation and thermocoagulation analyses were based on smaller exploratory subgroups of 5 and 6 patients, respectively. These results should therefore be interpreted as proof‐of‐concept associations rather than biomarker‐level validation, and larger, ideally prospective cohorts will be required to assess their clinical impact. Second, the comparison with the clinically defined SOZ should be interpreted as concordance with retrospective clinical SOZ assessment, not as independent SOZ validation. The clinical SOZ labels and the model‐derived metrics were both constrained by the same implanted sEEG coverage and derived within the same presurgical evaluation context, which limits the independence of this comparison. Responses to 50 Hz stimulation are difficult to evaluate in patients with very active interictal backgrounds, where frequent epileptiform discharges occur even without stimulation. Additionally, even though high fragility may suggest susceptibility to pathological state transitions, in principle, network instability could also lead to functional or healthy dynamic states, as seen in eloquent cortex.^43^ Therefore, weak or nonsignificant correlations with the clinical SOZ or stimulation response are not necessarily unexpected and do not reduce the potential utility of the metrics. Relatedly, a significant difference in metric values between SOZ and non‐SOZ channels does not automatically imply that channels can be reliably classified in a prospective way. Such interpretations are often confounded when combined with machine learning pipelines, where model performance depends on choice of classifying algorithms and where summary metrics like the area under the receiver operating curve (AUC‐ROC) may mask underlying uncertainty. To avoid this limitation, we intentionally did not include classification models or AUC analyses, as they can overstate per‐channel predictive utility. Despite this, higher fragility or source–sink values indicate increased probability of SOZ involvement and can inform clinical decisions, such as selecting channels for stimulation during presurgical evaluation.

The considered brain areas are not isolated systems and still receive inputs from unrecorded regions, which are not captured in our models. Electrode contacts may also lie in structurally or functionally distinct tissue types, including white matter or subcortical regions.^41^ Nevertheless, we showed that by including these areas and using a limited set of channels, clinically meaningful results can still be achieved. A more detailed analysis of how fragility and related measures depend on tissue type and anatomical location would be an important direction for future work. Longer‐term outcomes after thermocoagulation could not be systematically assessed, as most patients subsequently underwent resective surgery, making it impossible to attribute later clinical outcome specifically to thermocoagulation. A larger cohort of patients undergoing thermocoagulation alone, or a cohort whose resected brain areas are precisely mapped to the initial locations of sEEG contacts, would be valuable to evaluate whether predicted increases in network resilience after virtual removal correlate with clinical outcome.

The extremely high similarity of incoming and outgoing fragility, as well as of source and sink metrics, is largely due to the estimated network matrices being close to symmetric, *A*_*ij*_ *≈ A*_*ji*_. This near‐symmetry likely reflects the combination of short‐lag prediction and ridge‐regularized least‐squares fitting. At a 5 ms prediction step, consecutive preprocessed sEEG samples are highly similar, so the estimated matrices seem to be dominated by stable covariance‐like spatial structure rather than by strongly directed lagged interactions. This interpretation is also consistent with the fitted eigenvalue spectra, which mostly contain small real eigenvalues well inside the unit circle, that is, the instability boundary for discrete‐time evolution operators (see Supplementary material on frequency dependence of fragility). The weak frequency dependence of fragility can be understood in the same framework. In a discrete‐time model, complex eigenvalues encode oscillatory modes, with their angle corresponding to frequency, and their radius describing how close the mode is to the unit circle, that is, to the instability boundary. Changing the perturbation frequency thus corresponds to moving the tested instability point along the unit circle. Strong frequency dependence would be expected if the fitted matrices contained complex eigenvalues close to the unit circle at specific angles, because different perturbation frequencies would then selectively emphasize different oscillatory modes. In our fitted spectra, however, eigenvalues were mostly real, small and not forming near‐unstable complex‐conjugate pairs associated with specific rhythms. Fragility was thus dominated mainly by the spatial participation of channels in the effective network encoded by A, rather than by resonance with specific brain rhythms (see Supplementary materials for more details). This also explains the high correlation between fragility and frequency‐agnostic source–sink metrics, which summarize closely related spatial aspects of the fitted matrix. Future work should test whether richer Koopman‐inspired or nonlinear model‐estimation approaches, for example delay‐embedded dynamic mode decomposition or sparse nonlinear dynamical models,^38^, ^46^, ^47^, ^48^, ^49^ can better preserve oscillatory, nonlinear, and potentially asymmetric network structure, and thereby reveal stronger directional or frequency‐specific properties.

In summary, our results support the feasibility, interpretability, and potential clinical translation of model‐based interictal network analysis. We believe that several practical and technical aspects should be explicitly considered when these metrics are used, including their dependence on recording time, the choice among related metric definitions, and the consequences of the ridge‐regularized short‐window model‐estimation framework for directional and frequency‐specific interpretation. For this reason, we put effort into clarifying how data selection, metric choice, and modeling assumptions shape the resulting network readouts. Nevertheless, this framework has shown strong clinical value in previous work and yielded stable, clinically meaningful results in our data. The methods are compatible with existing sEEG recordings and offer interpretable, patient‐specific insights that may support SOZ assessment, improve the understanding of stimulation effects during presurgical evaluation, and motivate further development of virtual resection planning.

## AUTHOR CONTRIBUTIONS

T.D.: conceptualization, methodology (analytical and numerical), interpretation, visualization, writing (original draft). D.L., K.K., A.E.: clinical data acquisition and annotation, writing (review & editing). R.P. and L.I.: conceptualization, interpretation, funding acquisition, writing (review & editing). All authors read and approved the final manuscript.

## FUNDING INFORMATION

This work was funded by the Swiss National Science Foundation grant (SNF 197766, awarded to L.I. and R.P.), by the European Research Council (ERC) under the European Union Horizon 2020 research and innovation program (grant agreement no. 758604), by an ERC starting grant (ENTRAINER, awarded to R.P.), by an ETH Grant (ETH‐25 18–2, awarded to R.P.), by the Koetser Foundation research grant (awarded to T.D. and L.I.), and by the research grants from the Swiss Epilepsy Foundation.

## CONFLICT OF INTEREST STATEMENT

The authors declare no competing interests.

## Supporting information


**Data S1:** Supporting Information.

## Data Availability

The sEEG data supporting this study are not publicly available due to patient‐privacy restrictions and the terms of the ethics approval but are available from the corresponding author upon reasonable request subject to a data‐sharing agreement. The analysis code is openly available at https://github.com/Swiss‐Epilepsy‐Center/epinetmap (release v1.0.1).
