# Supplementary material for: Bridging computational and clinical strategies for presurgical identification of epileptogenic networks
Source: Epilepsia Open. 2026 Jul 24:10.1002/epi4.70311. Online ahead of print. doi: 10.1002/epi4.70311 (PMC13397325; doi:10.1002/epi4.70311)
Supplement: Supplementary file 1 — Data S1: Supporting Information. [file EPI4-9999-0-s001.zip › epi470311-sup-0002-Supinfo2.pdf]

Frequency dependence of fragility

To assess whether fragility estimates were driven by a specific oscillatory frequency, we computed a frequency-specific normalized incoming and outgoing fragility. Across patients, the dependence on frequency was weak and smooth. Importantly, the relative ordering of channels was preserved across frequencies: channels with high fragility at low frequencies remained fragile channels at higher frequencies. This led to a frequency-independent spatial profile of fragility.

This behavior is consistent with the spectra of the fitted linear models. For a discrete-time model, $x(t+1) = Ax(t)$, fragility at frequency f tests perturbations that move the system toward the unit-circle point $z(f) = e^{i\omega} = e^{i2\pi f/f_s}$.

The frequency dependence enters through the factor $(A - e^{i\omega}I)^{-1}$. For a diagonalizable matrix,

$$(A - e^{i\omega}\,\mathbb{1})^{-1} = \sum_m \frac{v_m u_m^T}{\lambda_m - z\,\mathbb{1}},$$

where λ_m are eigenvalues and v_m and u_m are right and left eigenvectors.

Strong frequency dependence would be expected if some eigenvalues were close to the unit circle at specific angles, because changing f would then selectively emphasize different oscillatory modes. In the fitted models, however, the eigenvalues are mostly real and with a small norm. Therefore, changing $z(f)$ across frequencies (corresponding here, with $f_s = 200$ Hz, to moving from z=1 to z=−1, i.e. over half of the unit circle) does not strongly reweight the modes. Fragility is dominated mainly by the spatial participation of nodes in the effective network, rather than by resonance with a specific frequency. We note that the small but stronger smooth changes at the boundaries of the tested frequency range are expected from the discrete-time mapping.

Taken together, the weak frequency dependence likely reflects the chosen model-estimation procedure: a short-window least-squares linear fit with strong ridge regularization. This fitting strategy favors stable, robust effective network estimates, but does not strongly preserve oscillatory near-instabilities in the fitted matrix A. In the present work, we intentionally kept the original metric definitions and model-estimation framework, because these metrics were previously shown in a large cohort to identify the seizure onset zone and predict outcome after resective surgery[1]. Future work should test whether alternative ways of estimating the linear network model, designed to better preserve oscillatory dynamics, can capture frequency-specific stability properties of epileptic networks and further improve clinically relevant predictions. For example, relying on delay-embedded dynamical mode decomposition to extract the dominant coherent modes of the network dynamics[2].

1. Li A, Huynh C, Fitzgerald Z, et al. Neural fragility as an EEG marker of the seizure onset zone. *Nat Neurosci*. 2021;24(10):1465-1474. doi:10.1038/s41593-021-00901-w

2. Dubcek T, Ledergerber D, Thomann J, et al. Electroencephalography-driven brain-network models for personalized interpretation and prediction of neural oscillations. *Clin Neurophysiol*. 2025;174:1-9. doi:10.1016/j.clinph.2025.03.030

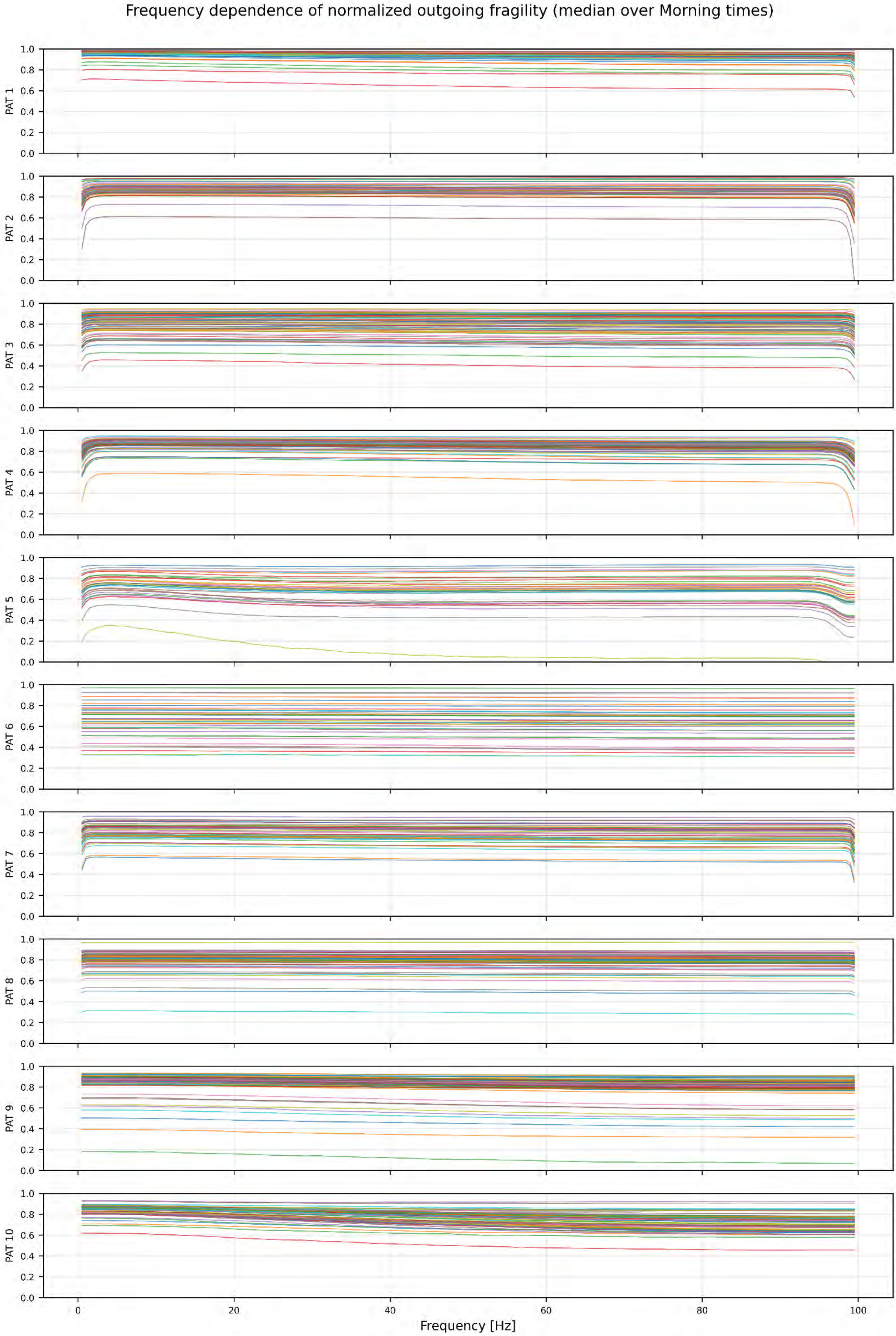
Frequency dependence of normalized outgoing fragility (median over Morning times)
PAT 1
PAT 2
PAT 3
PAT 4
PAT 5
PAT 6
PAT 7
PAT 8
PAT 9
PAT 10
0.0
0.2
0.4
0.6
0.8
1.0
0
20
40
60
80
100
Frequency [Hz]

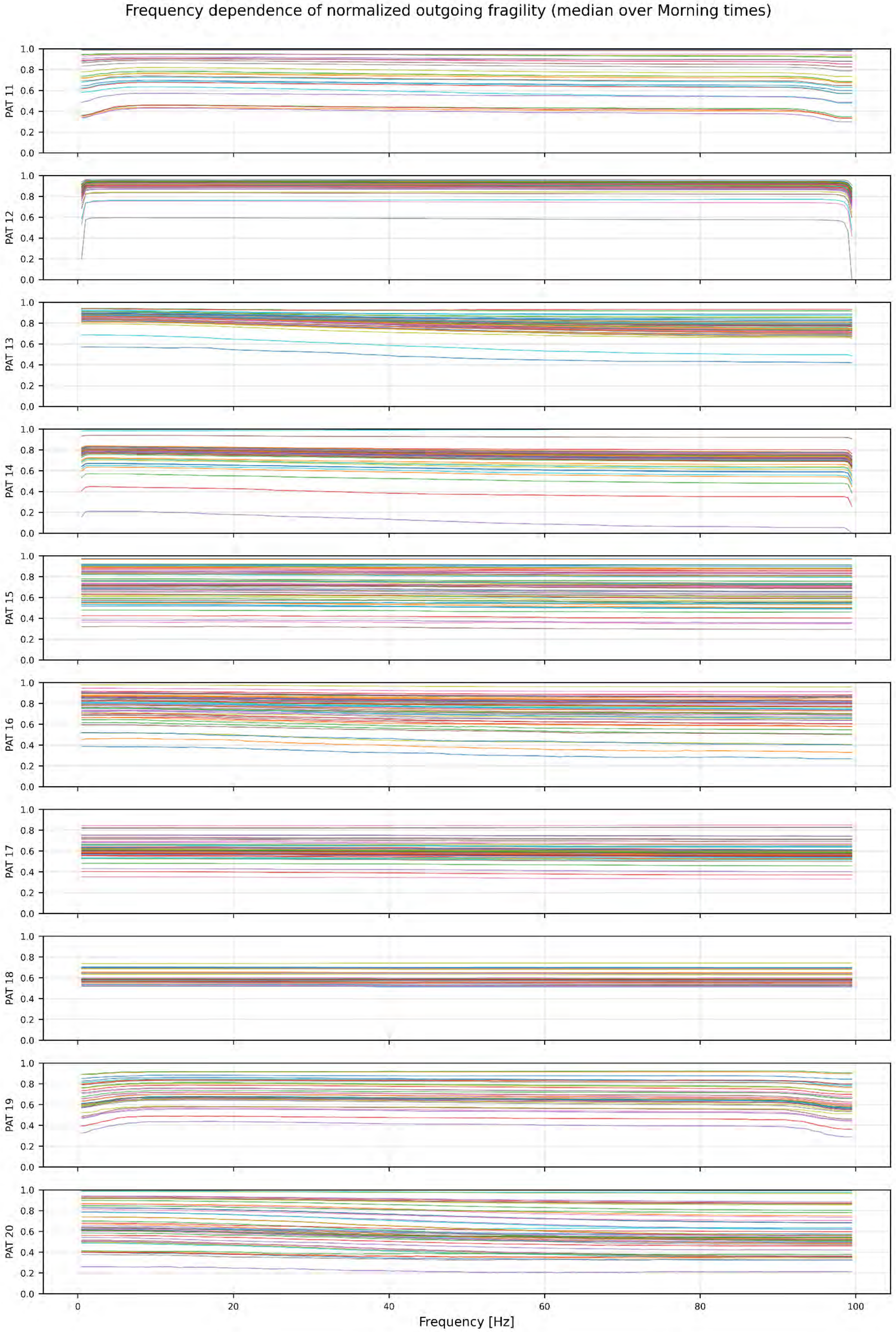

Frequency dependence of normalized outgoing fragility (median over Morning times)
PAT 11
PAT 12
PAT 13
PAT 14
PAT 15
PAT 16
PAT 17
PAT 18
PAT 19
PAT 20
0.0
0.2
0.4
0.6
0.8
1.0
0
20
40
60
80
100
Frequency [Hz]

A eigenvalues at random time points in Morning

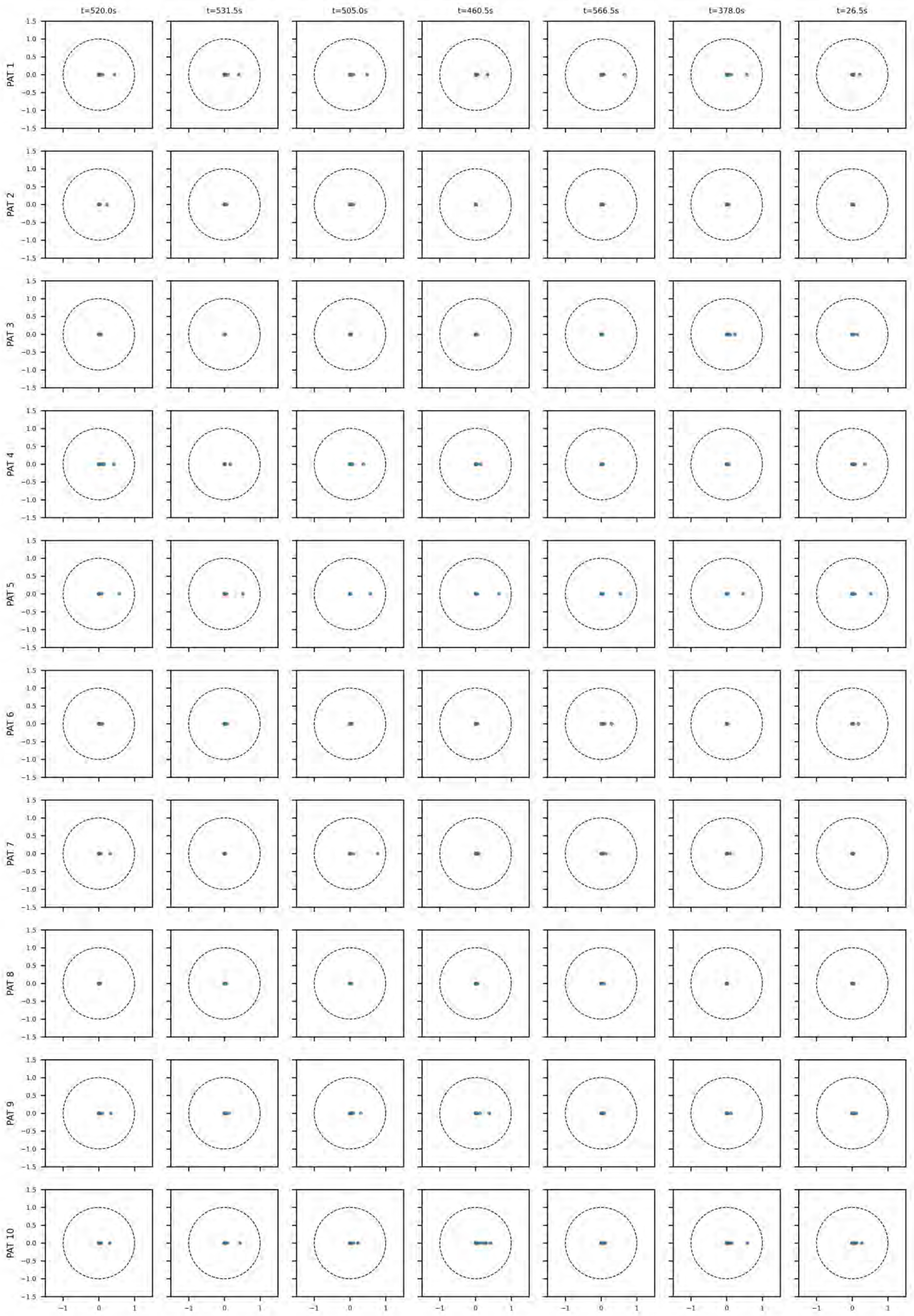

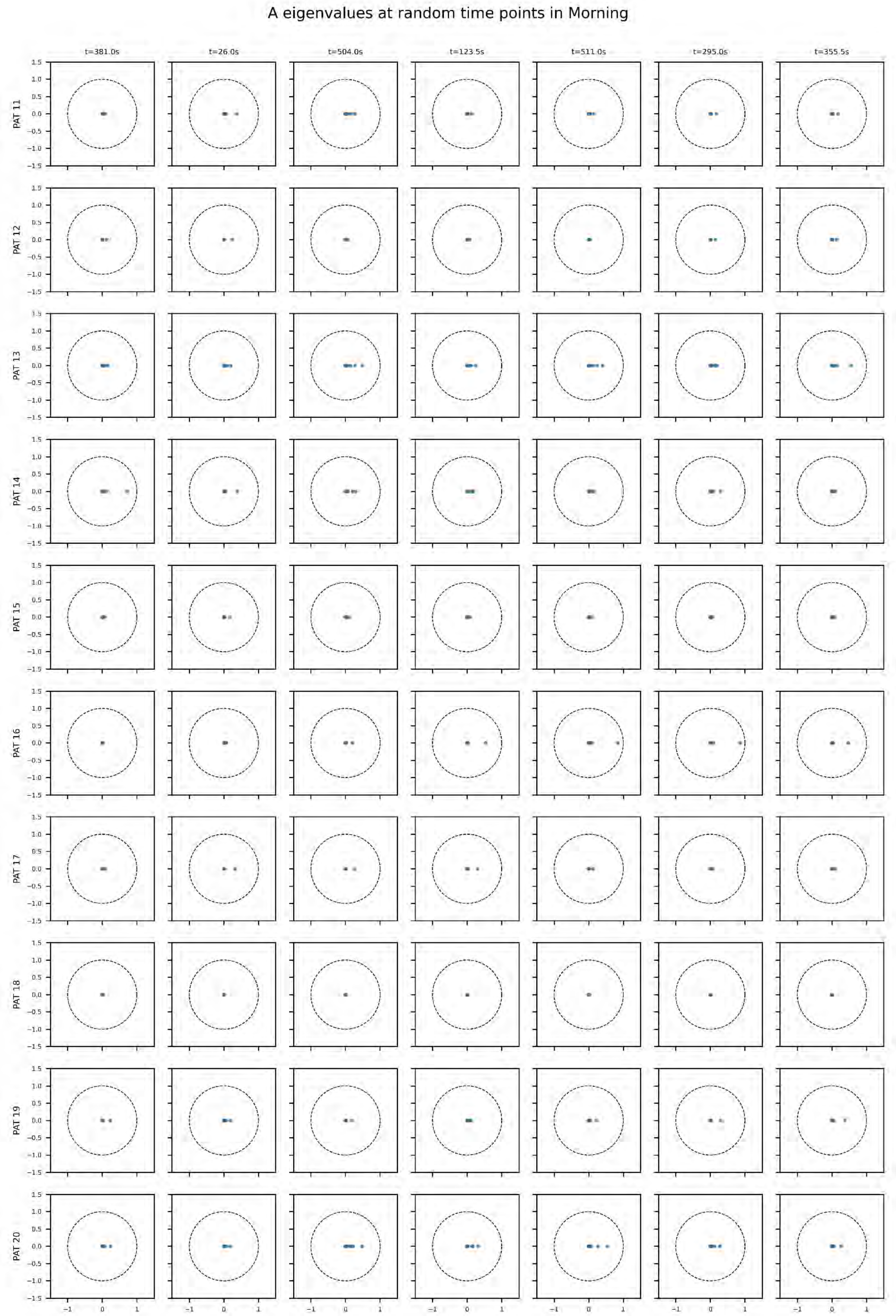
A eigenvalues at random time points in Morning
t=381.0s
t=26.0s
t=504.0s
t=123.5s
t=511.0s
t=295.0s
t=355.5s
PAT 11
PAT 12
PAT 13
PAT 14
PAT 15
PAT 16
PAT 17
PAT 18
PAT 19
PAT 20
