# Supplementary figures and images for: Bridging computational and clinical strategies for presurgical identification of epileptogenic networks
Source: Epilepsia Open. 2026 Jul 24:10.1002/epi4.70311. Online ahead of print. doi: 10.1002/epi4.70311 (PMC13397325; doi:10.1002/epi4.70311)

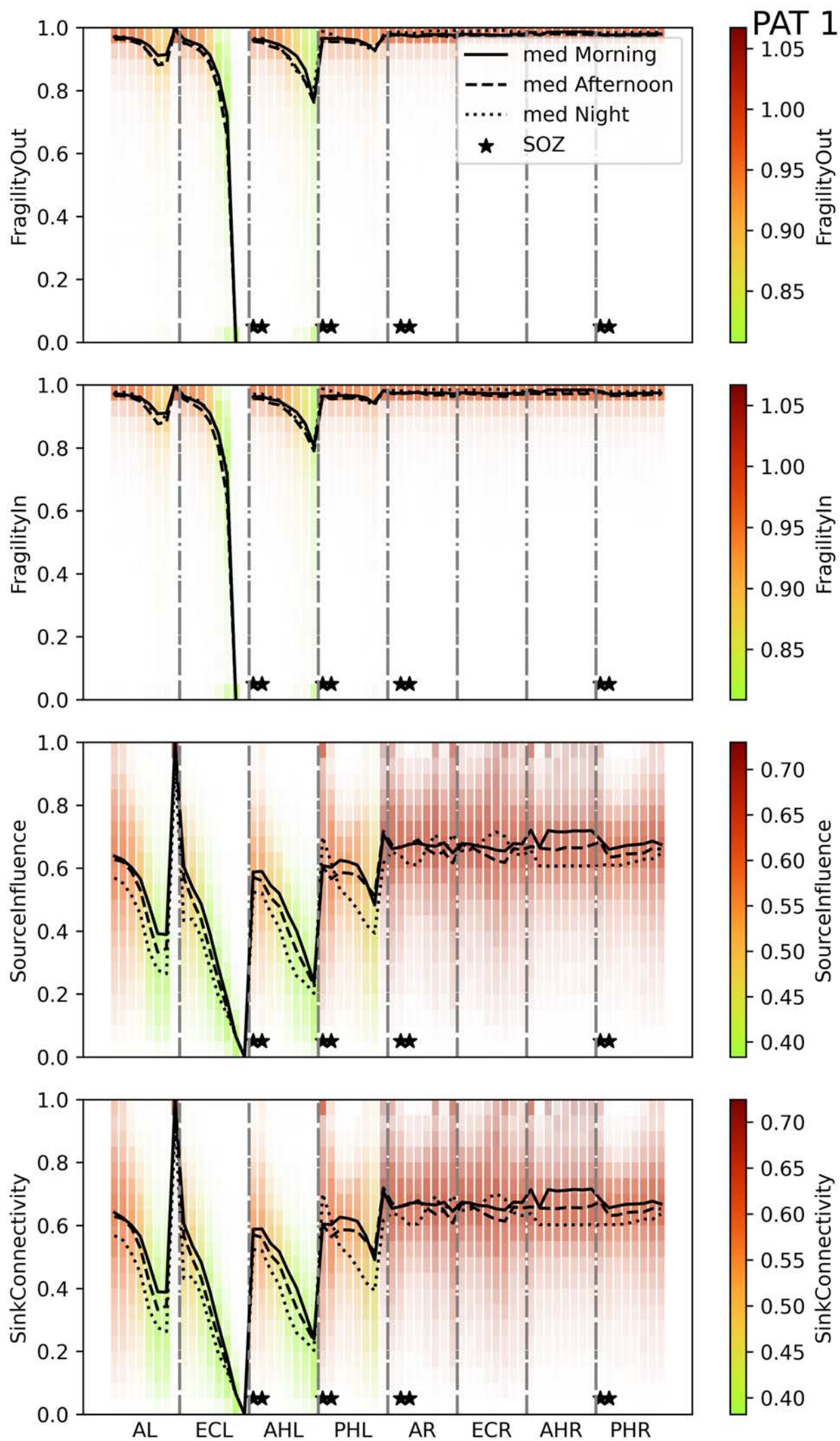

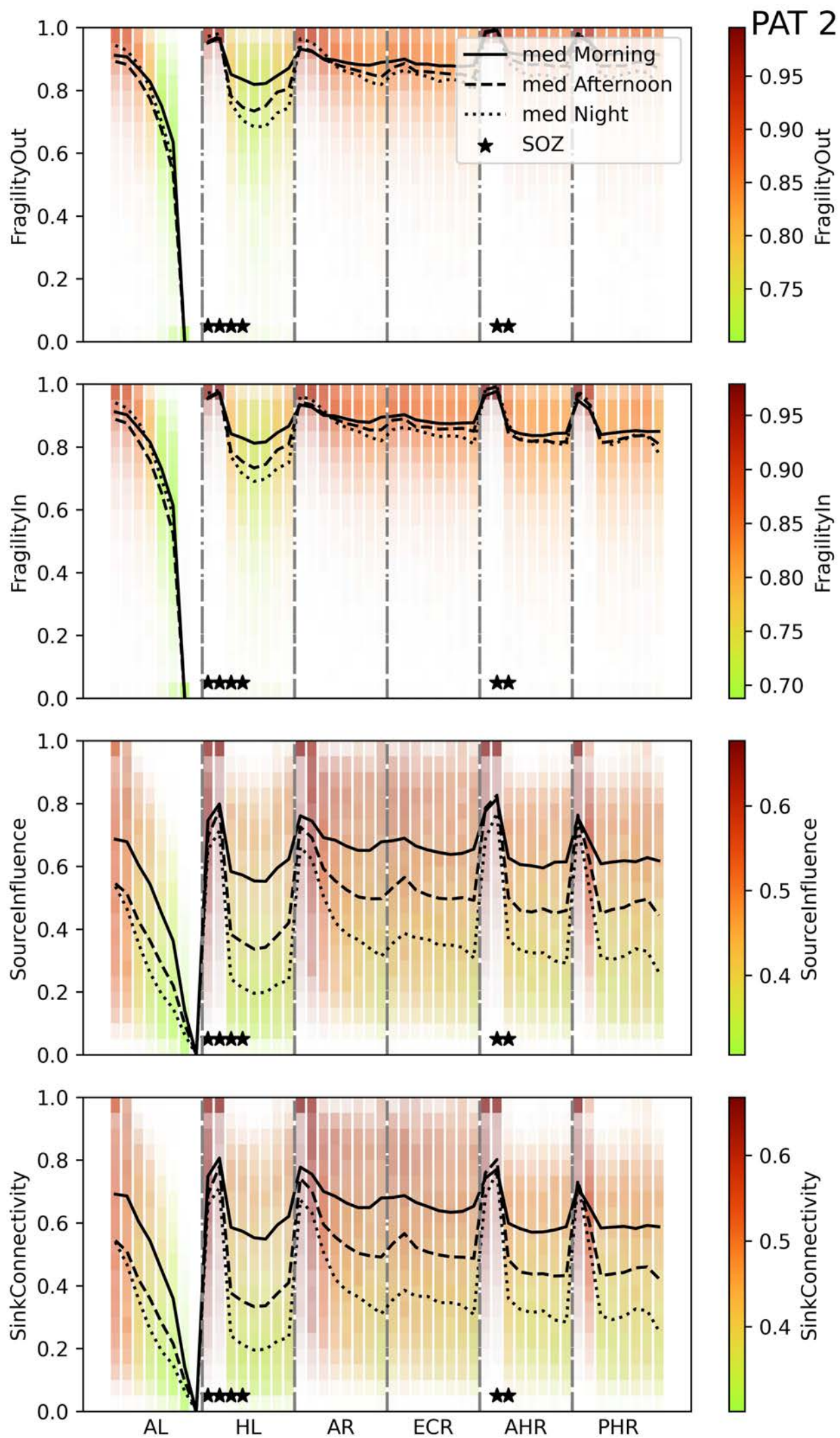

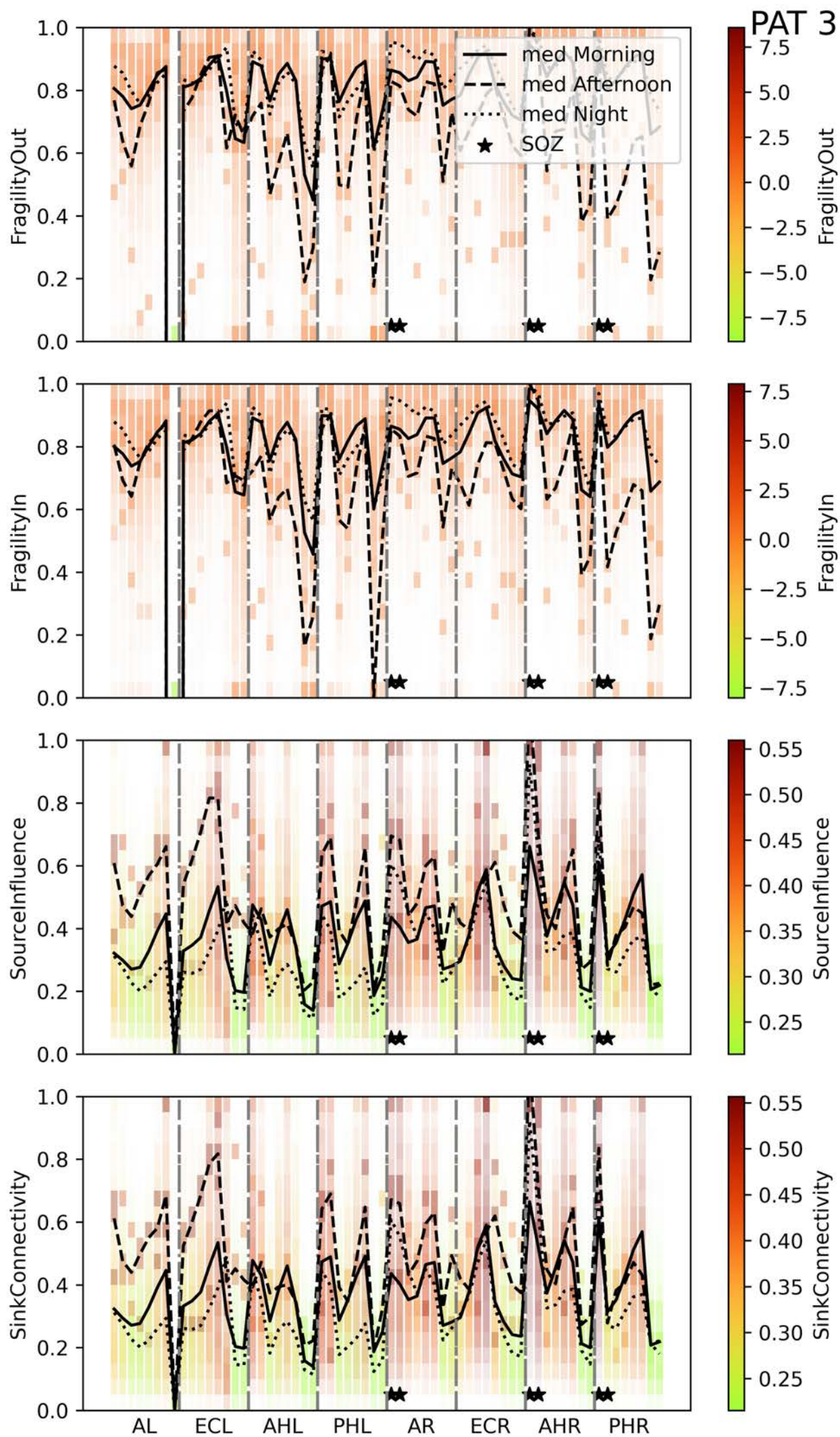

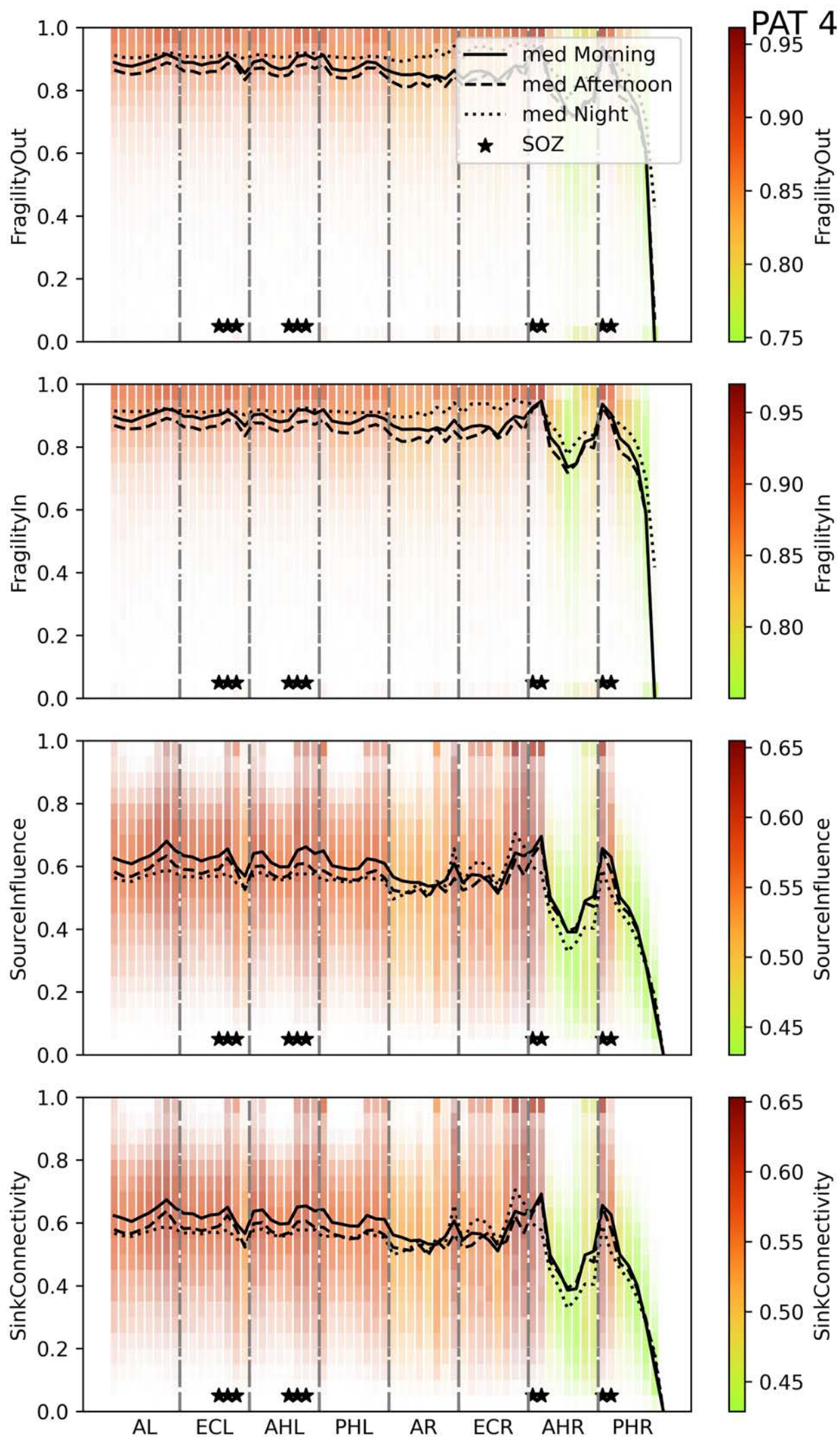

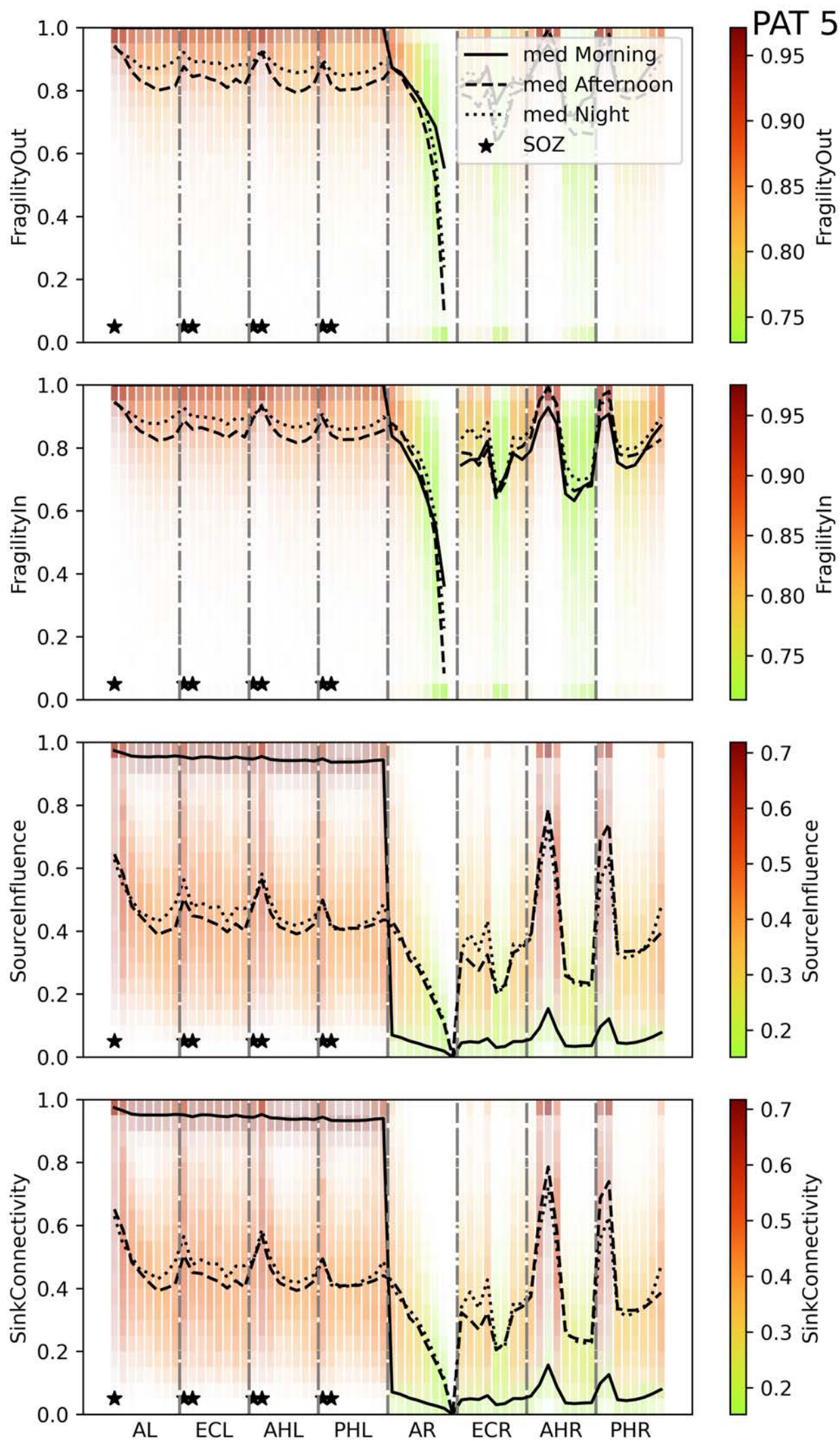

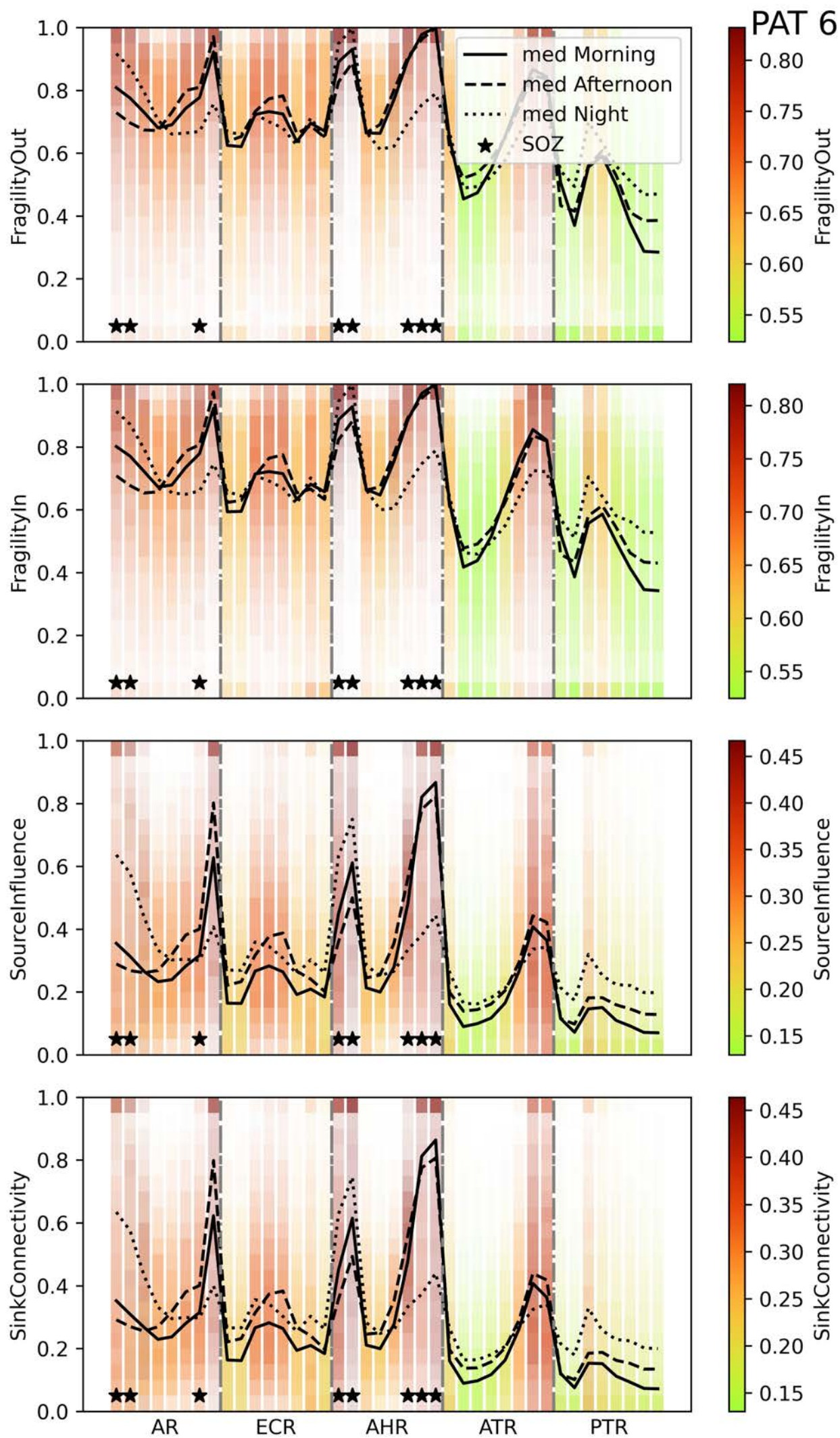

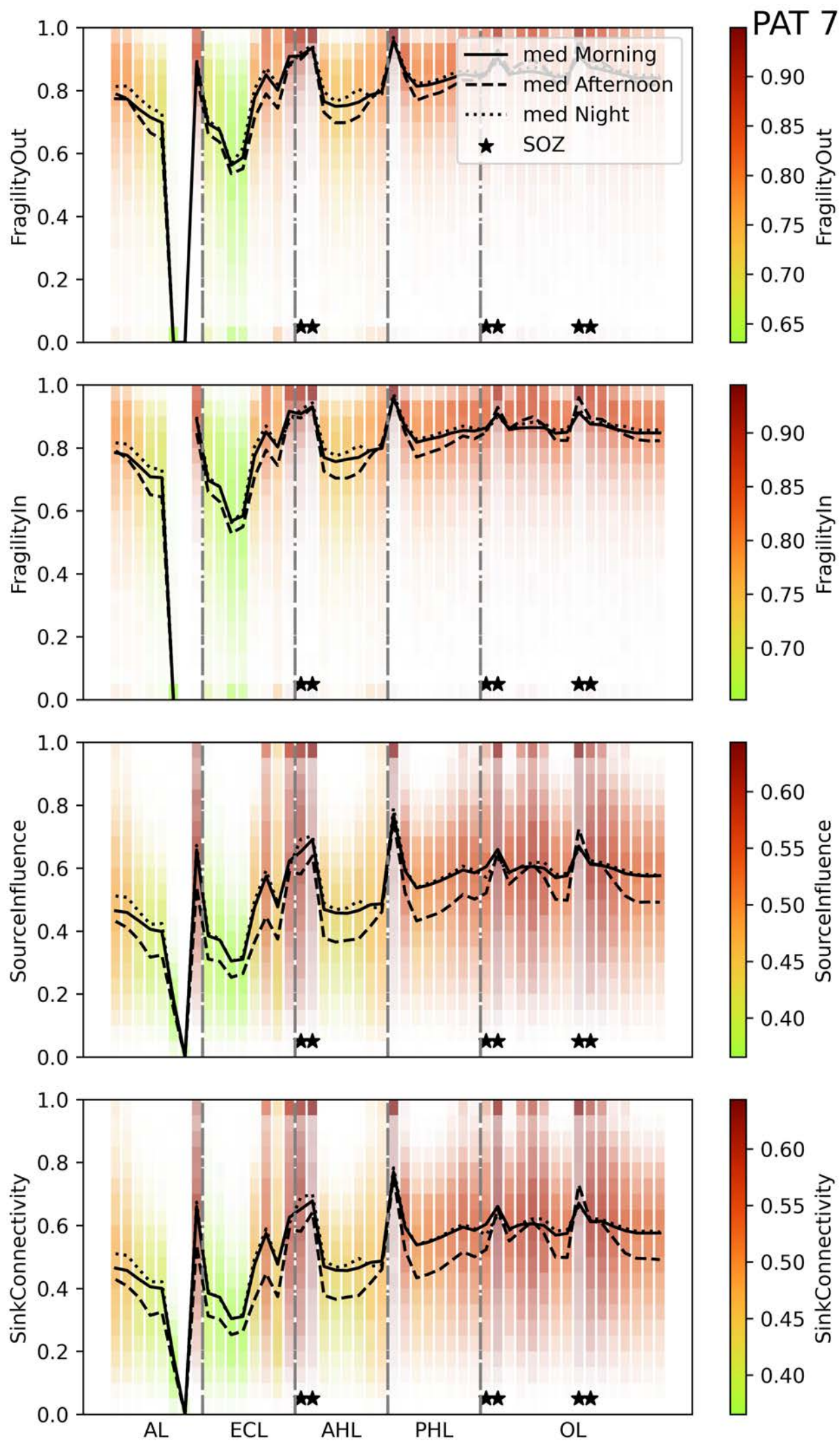

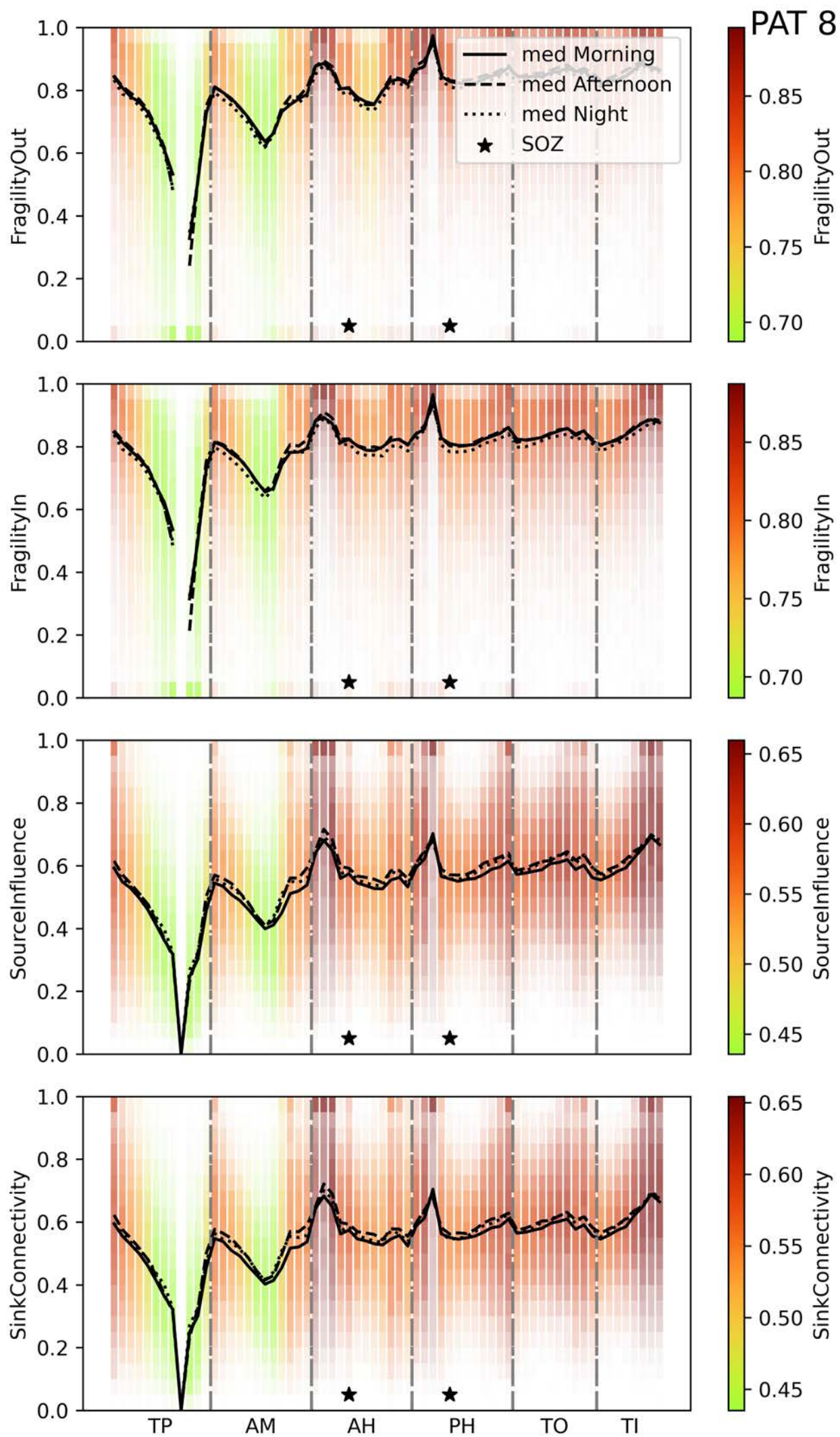

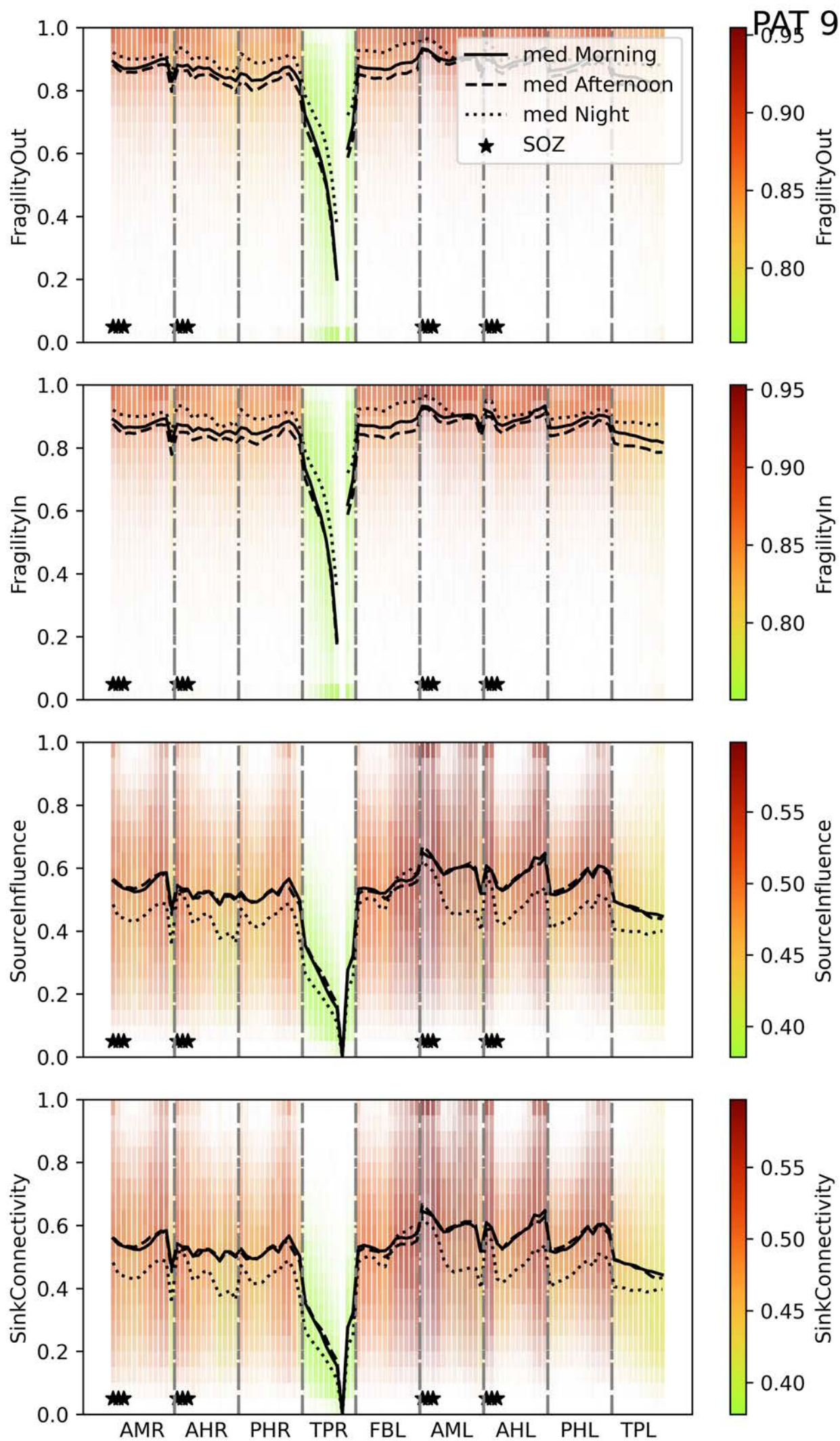

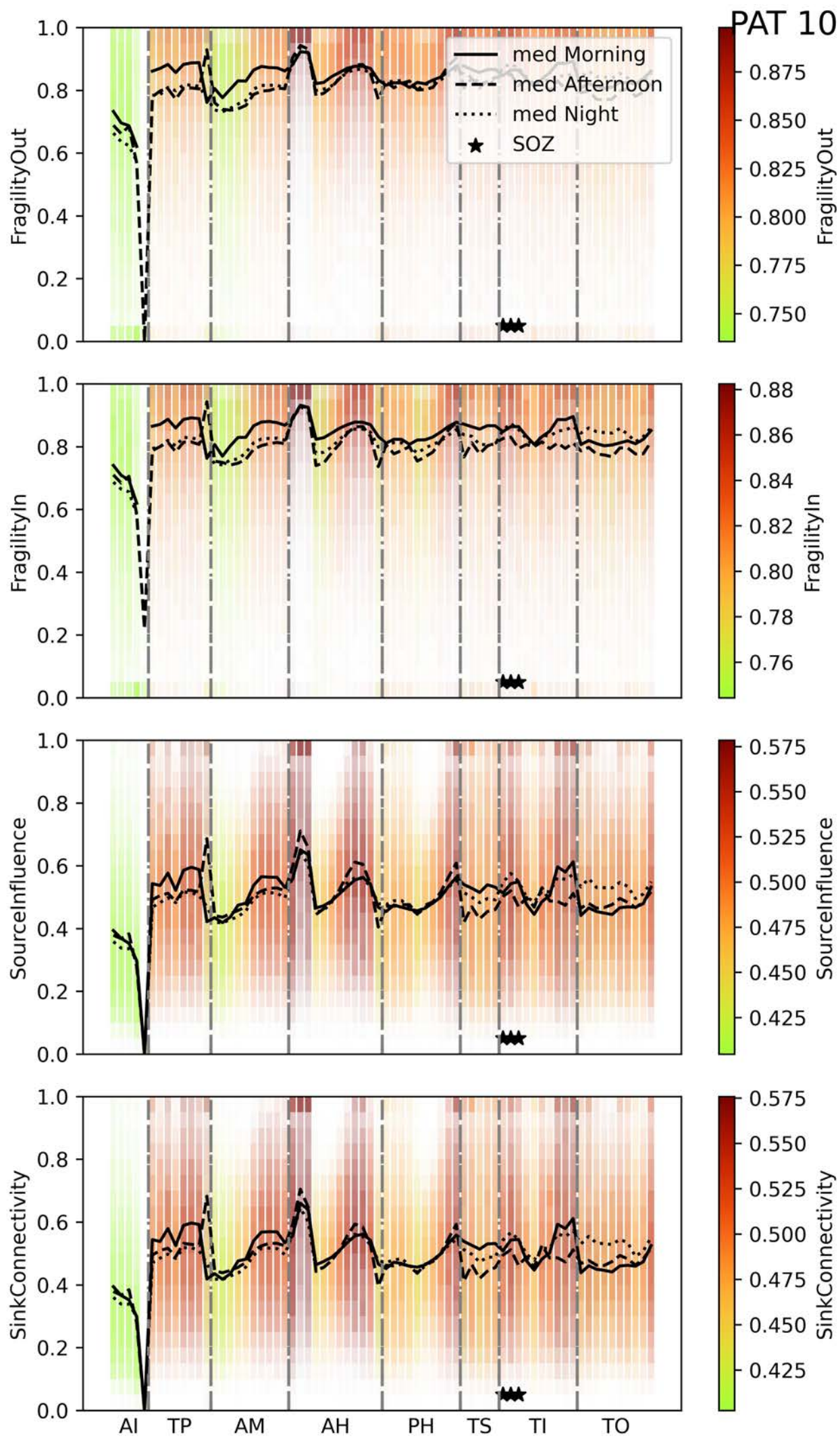

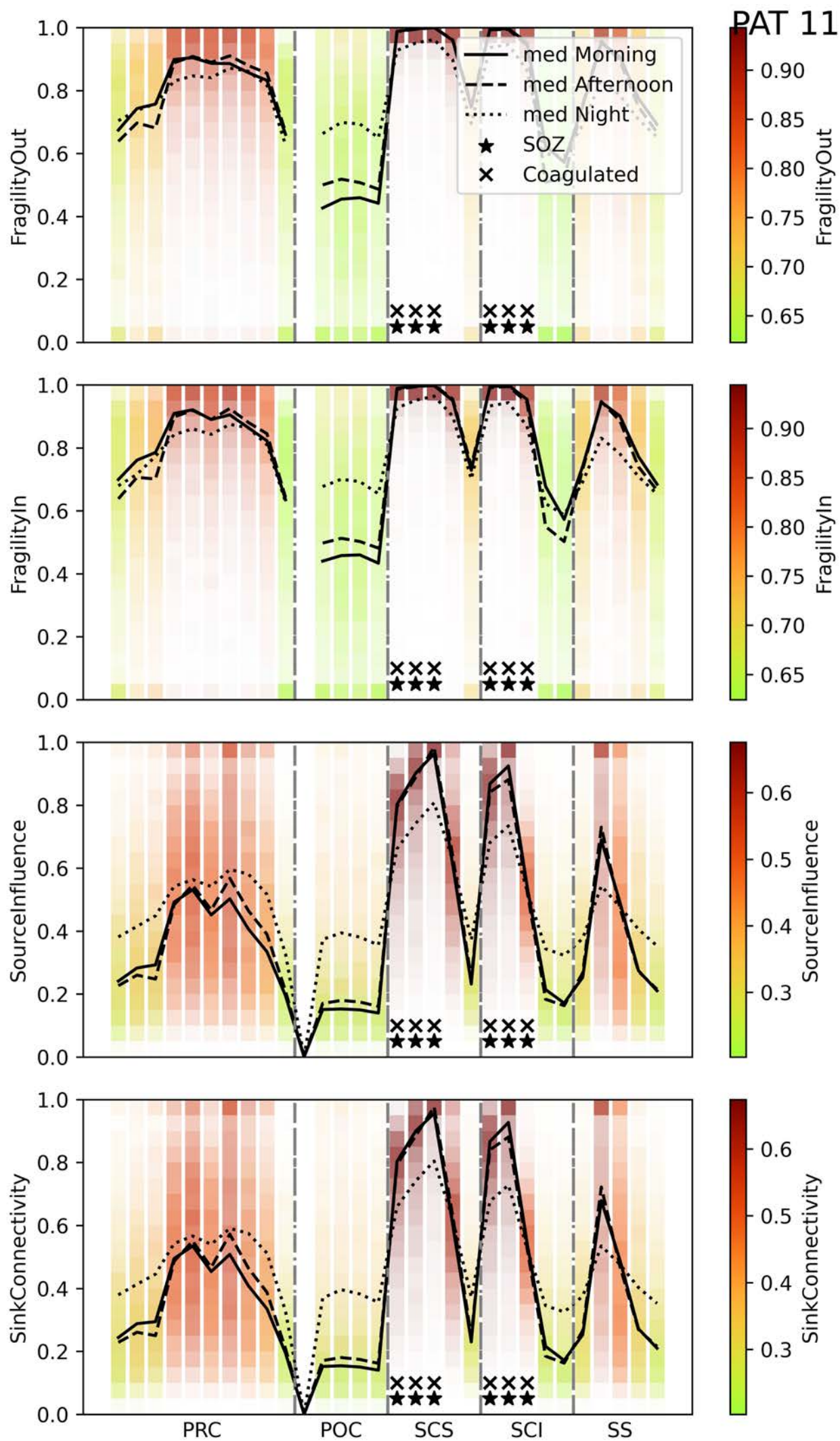

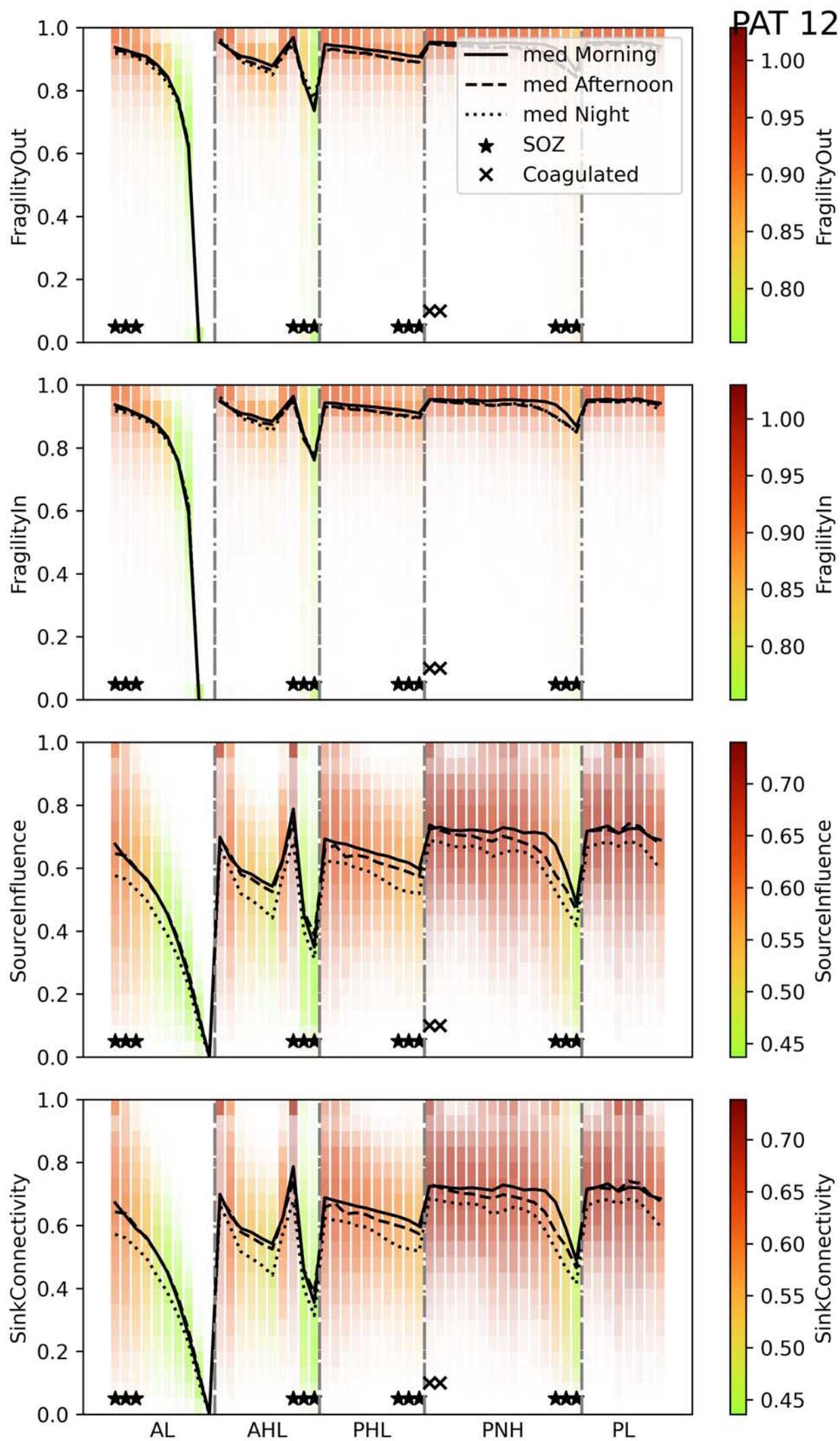

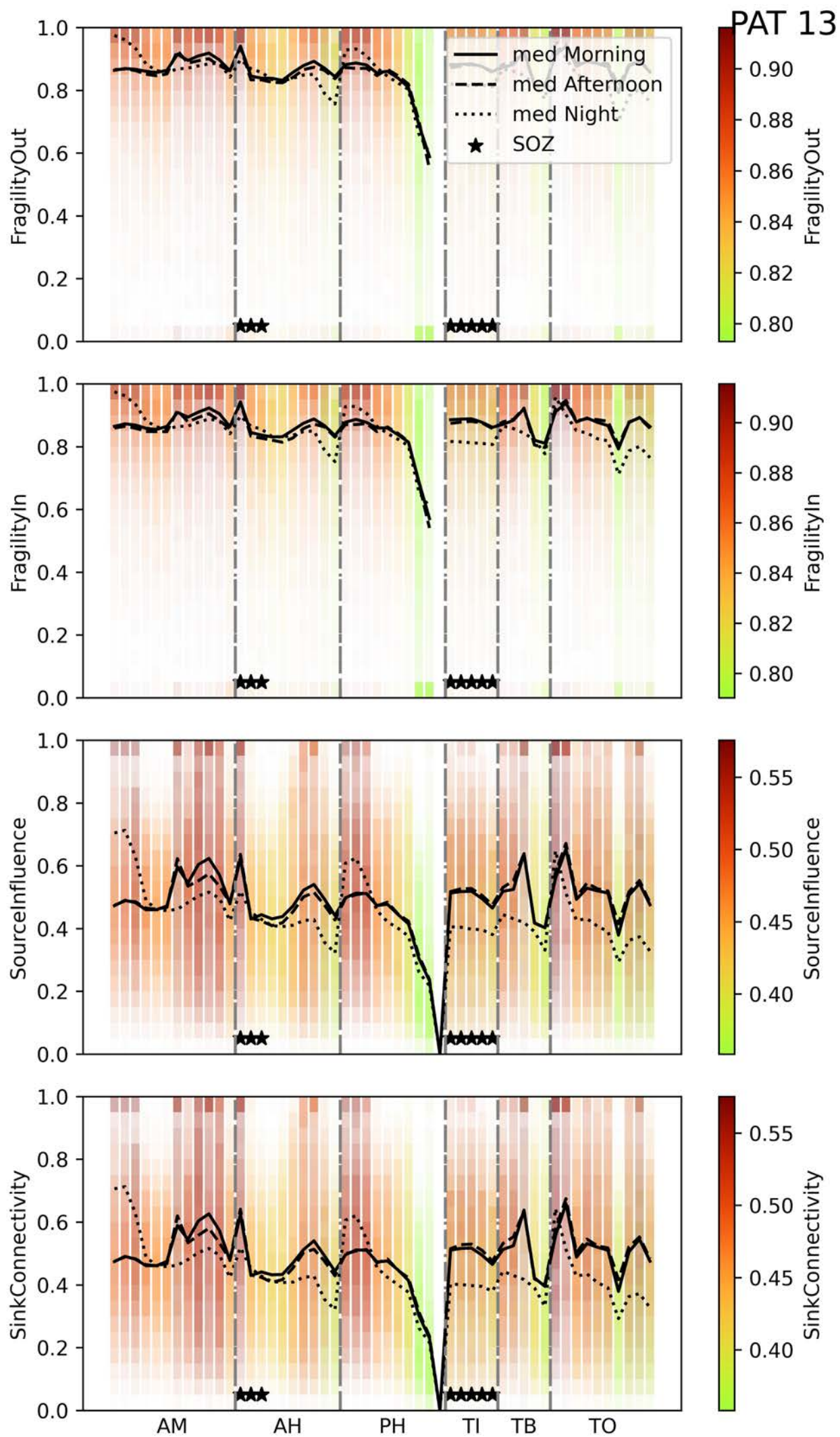

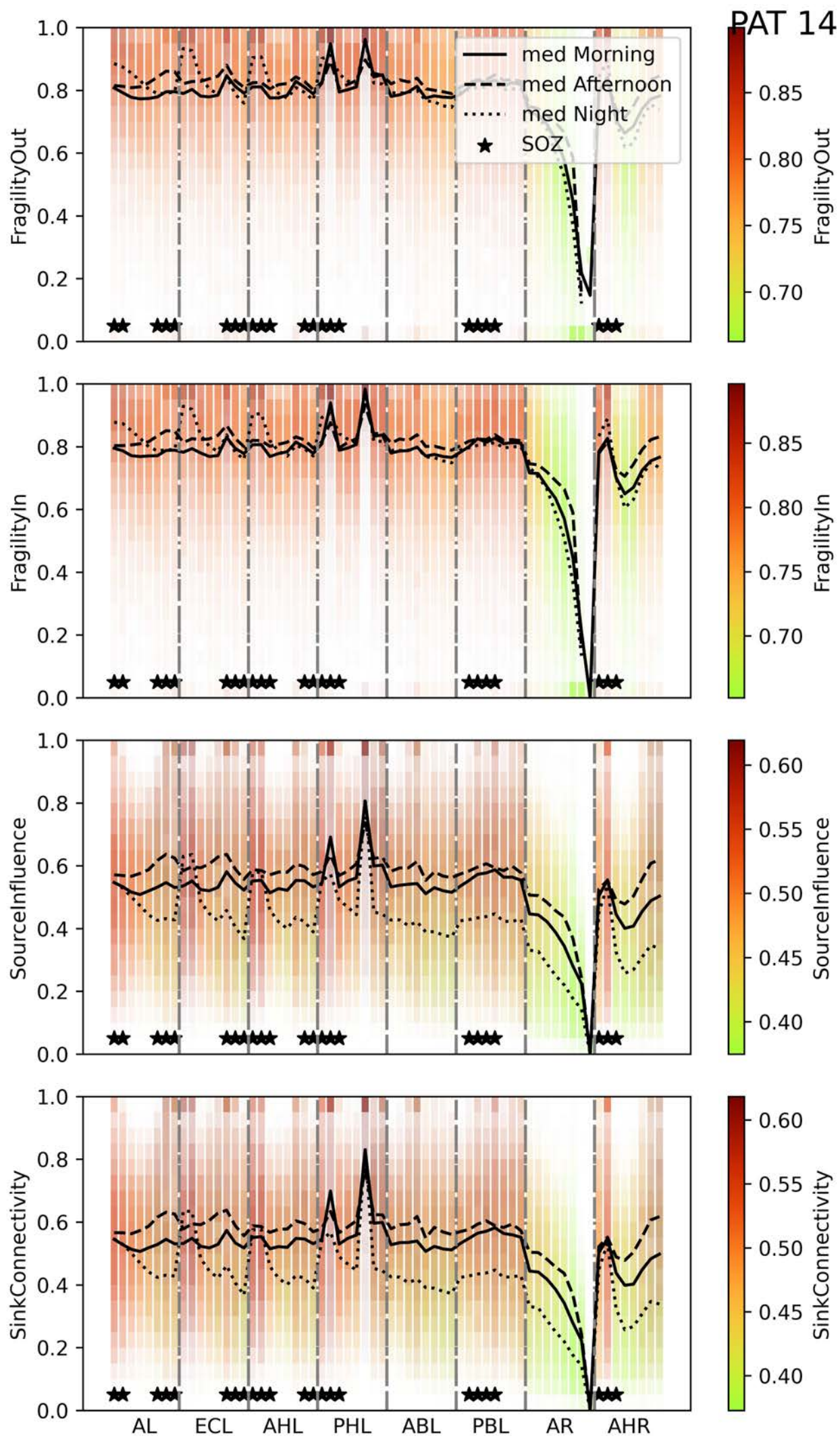

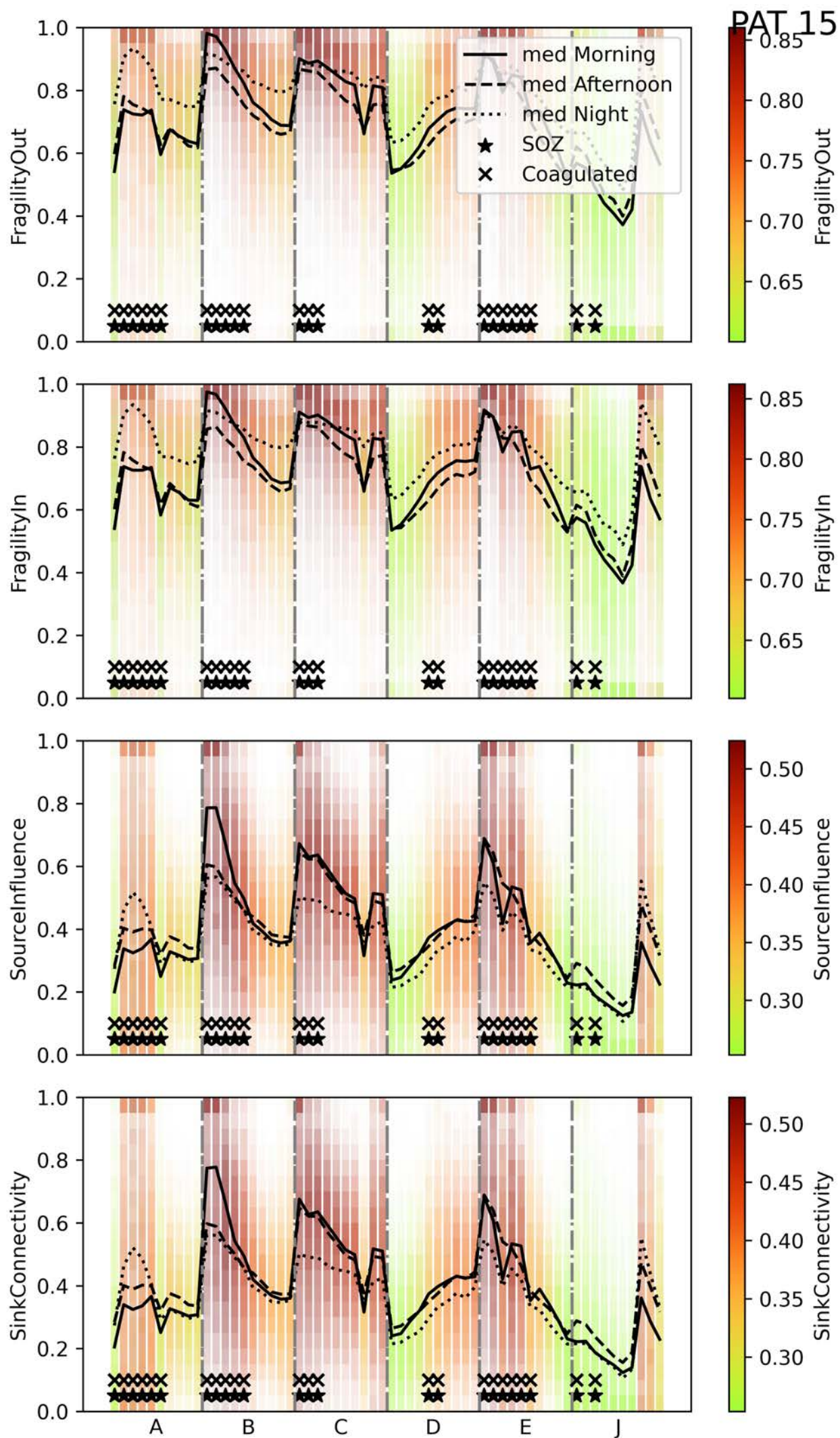

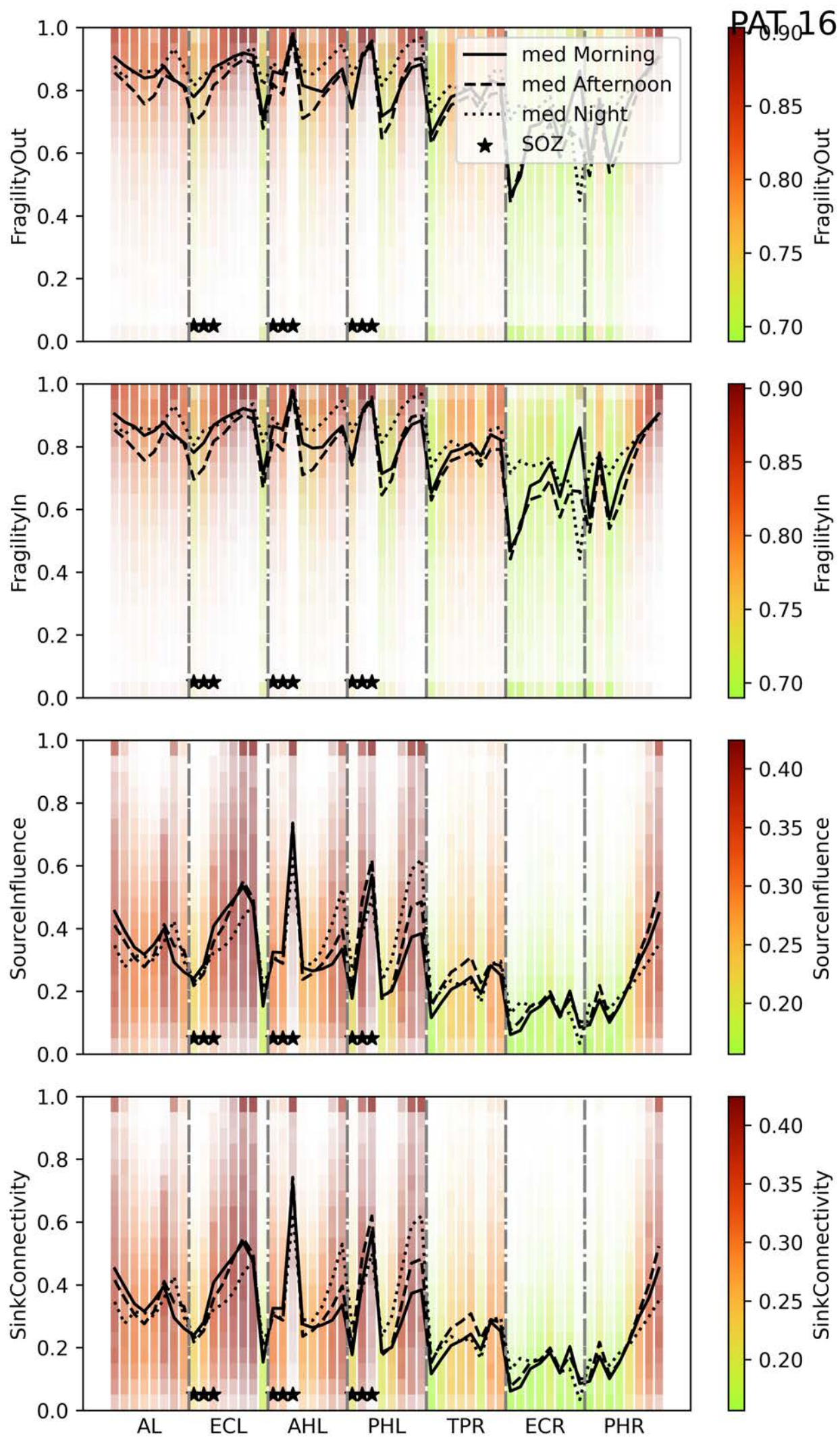

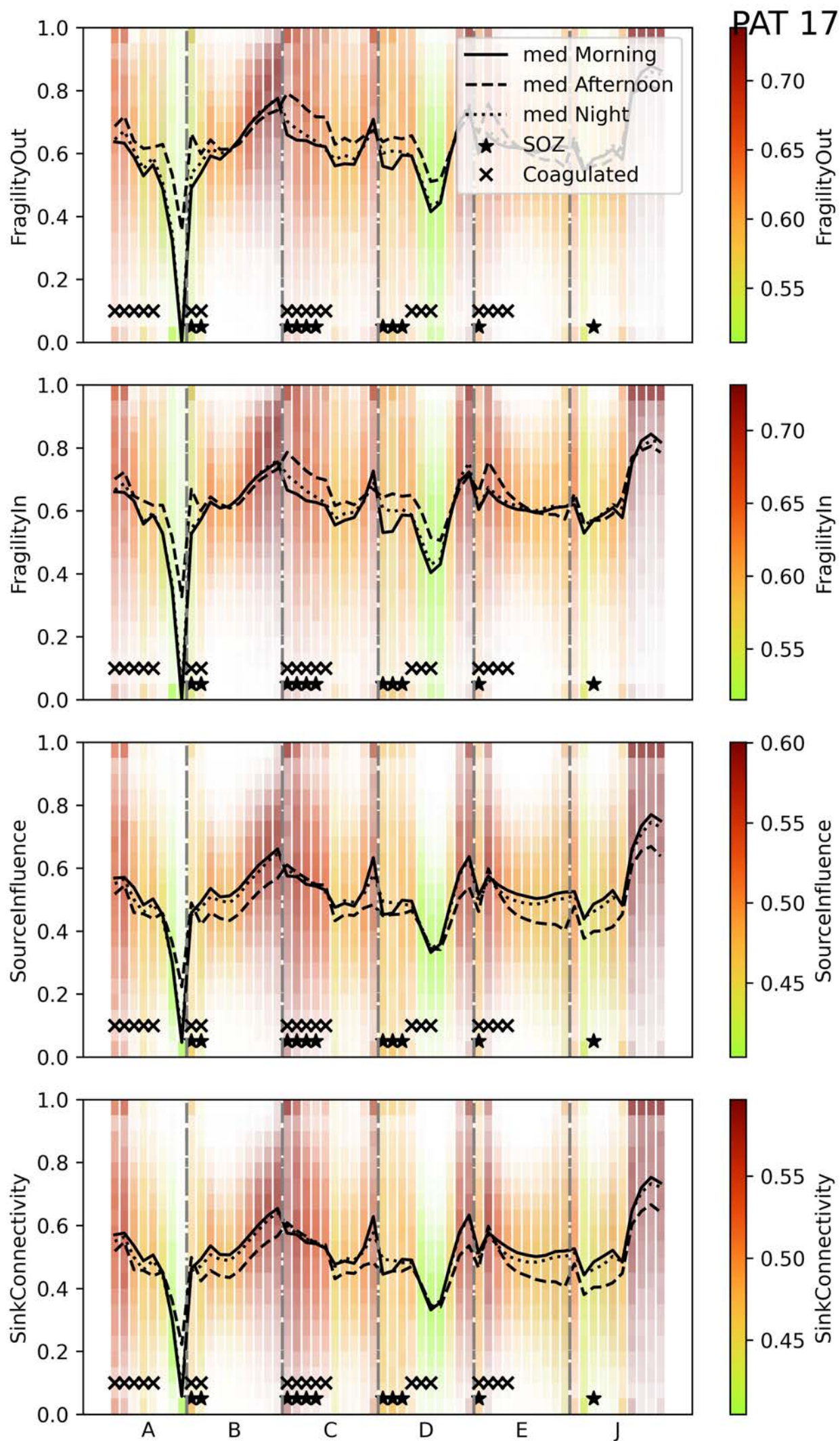

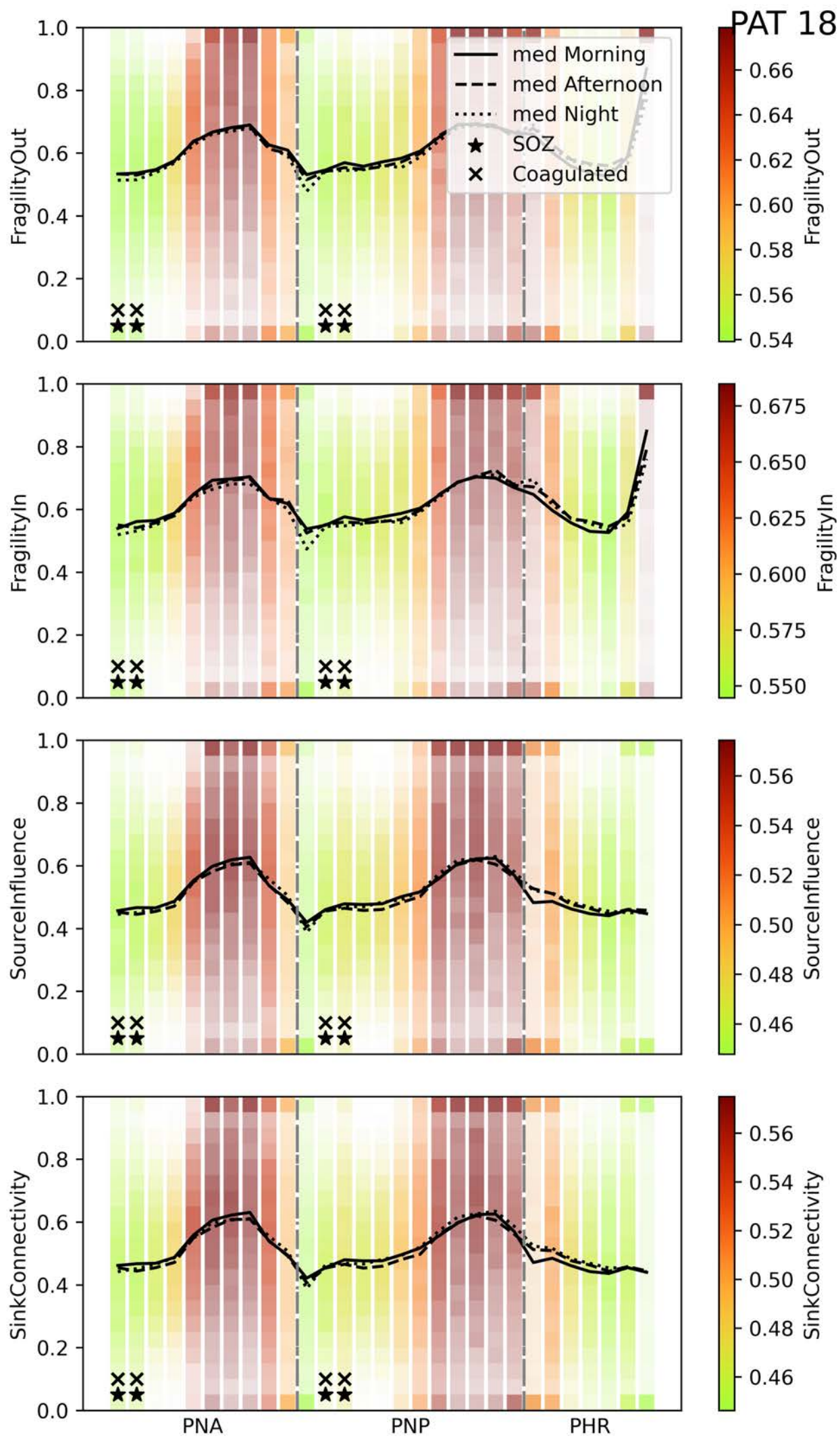

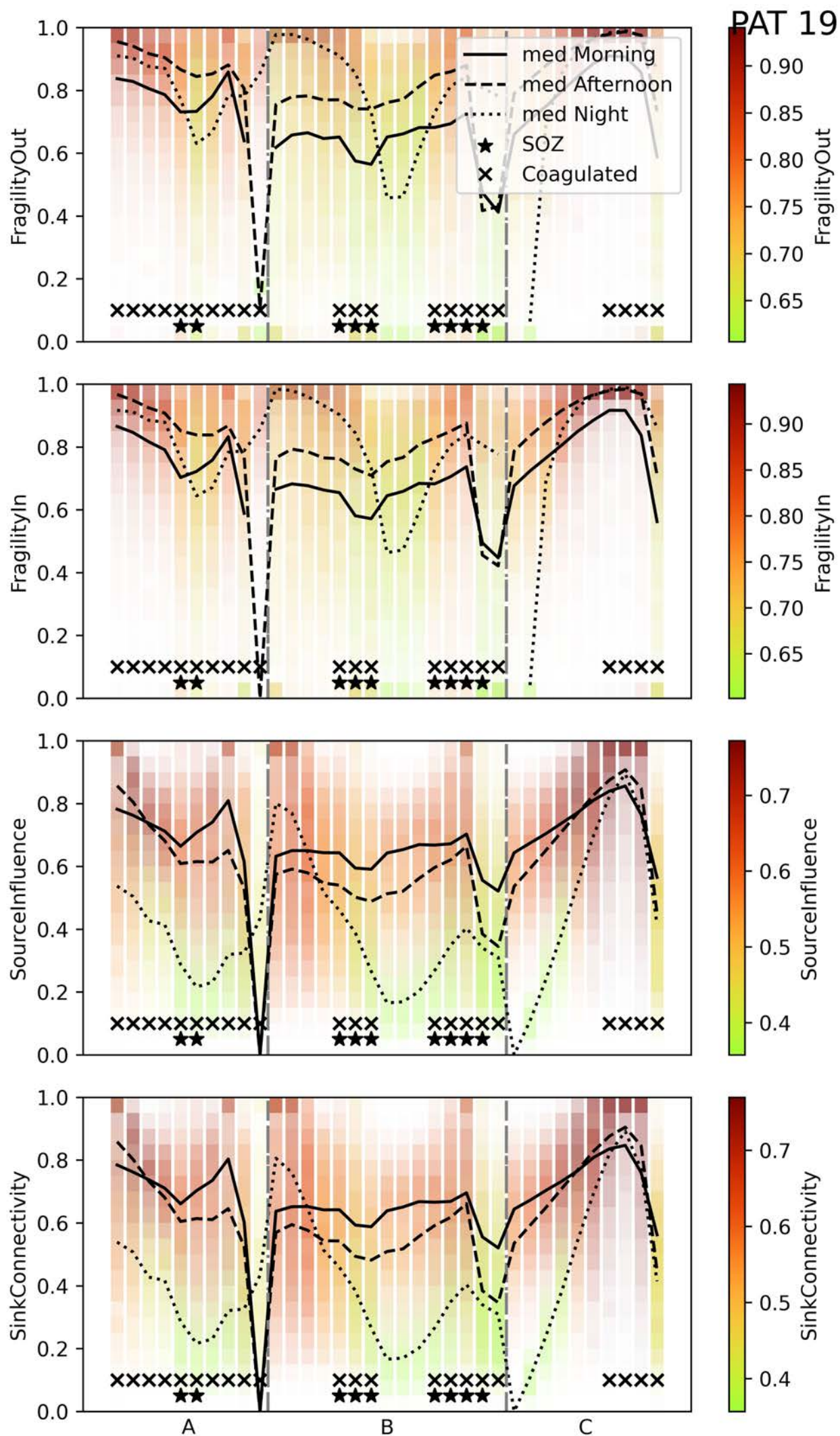

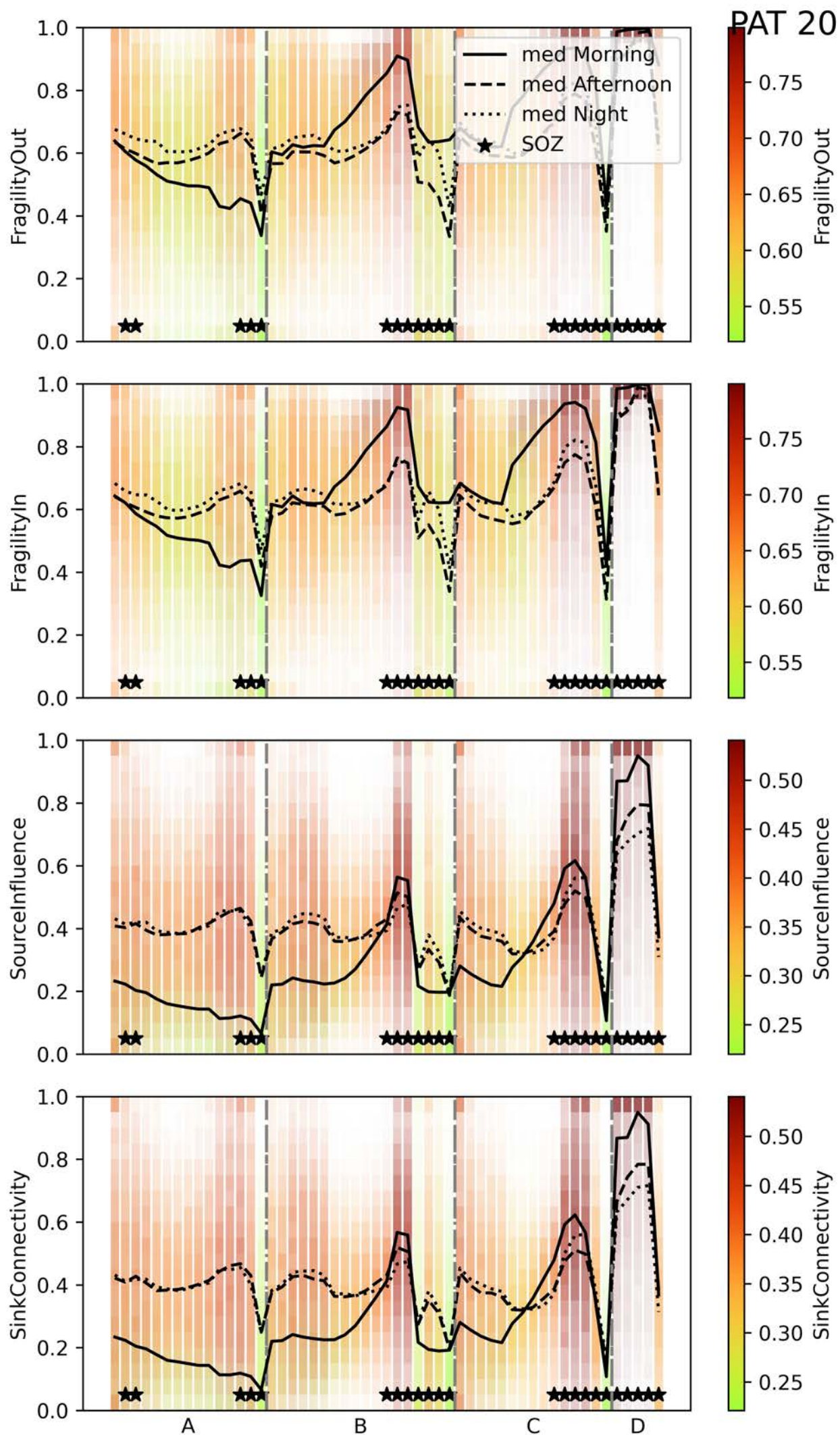

Supplement: Supplementary file 1 — Data S1: Supporting Information. [file EPI4-9999-0-s001.zip › epi470311-sup-0003-Supinfo3.pdf]

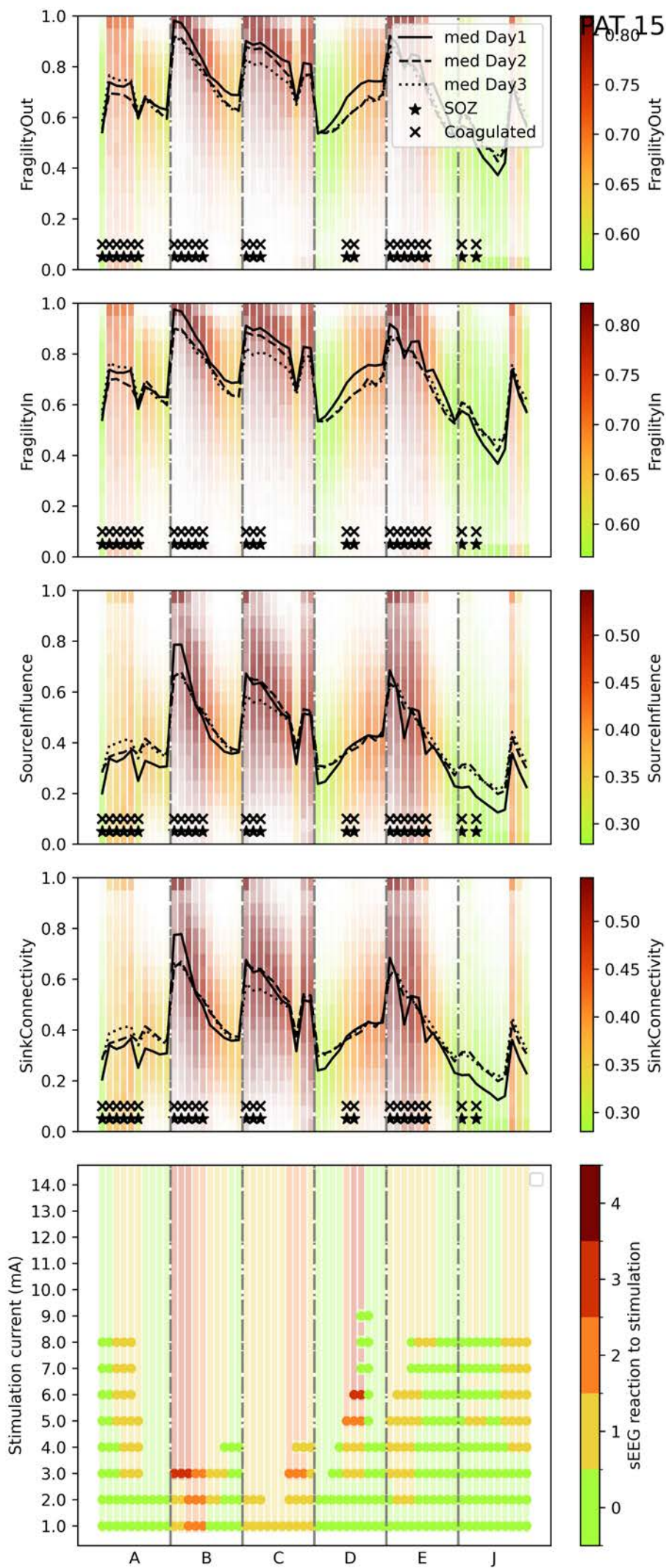

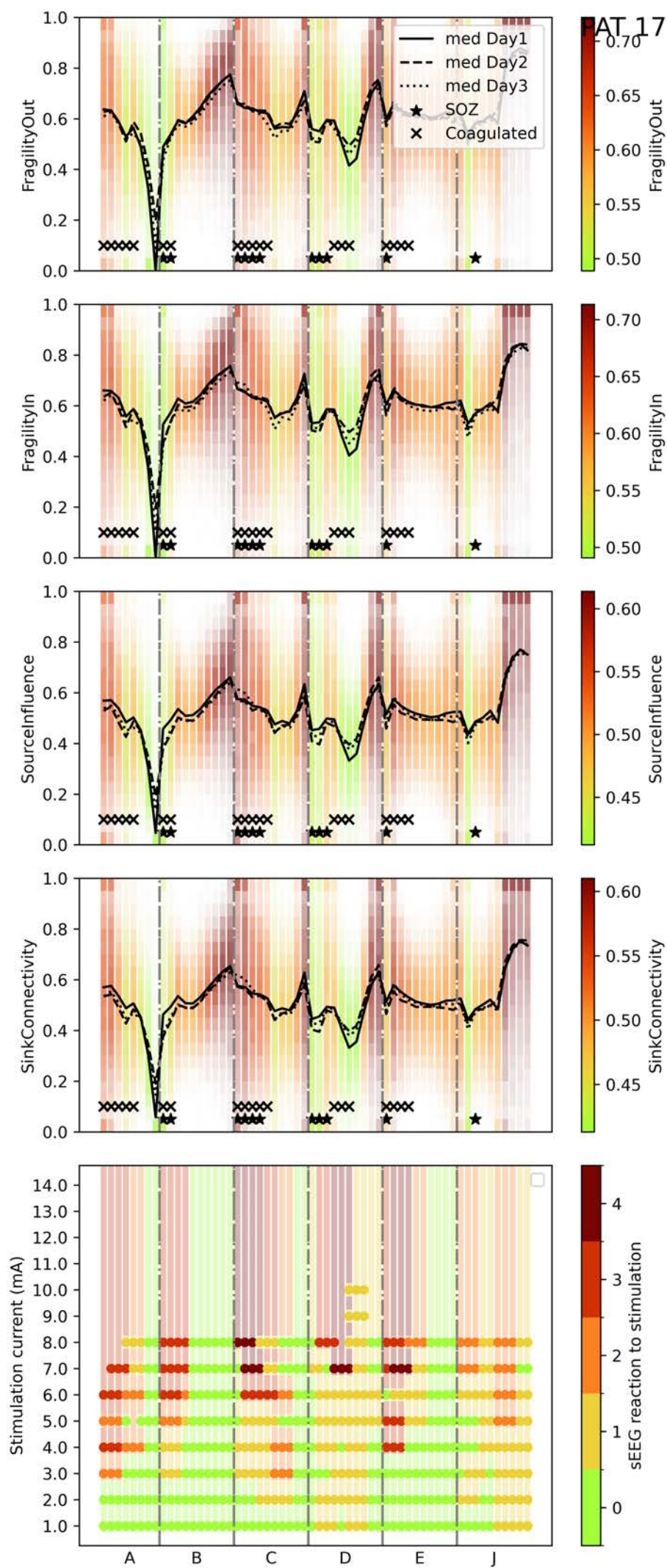

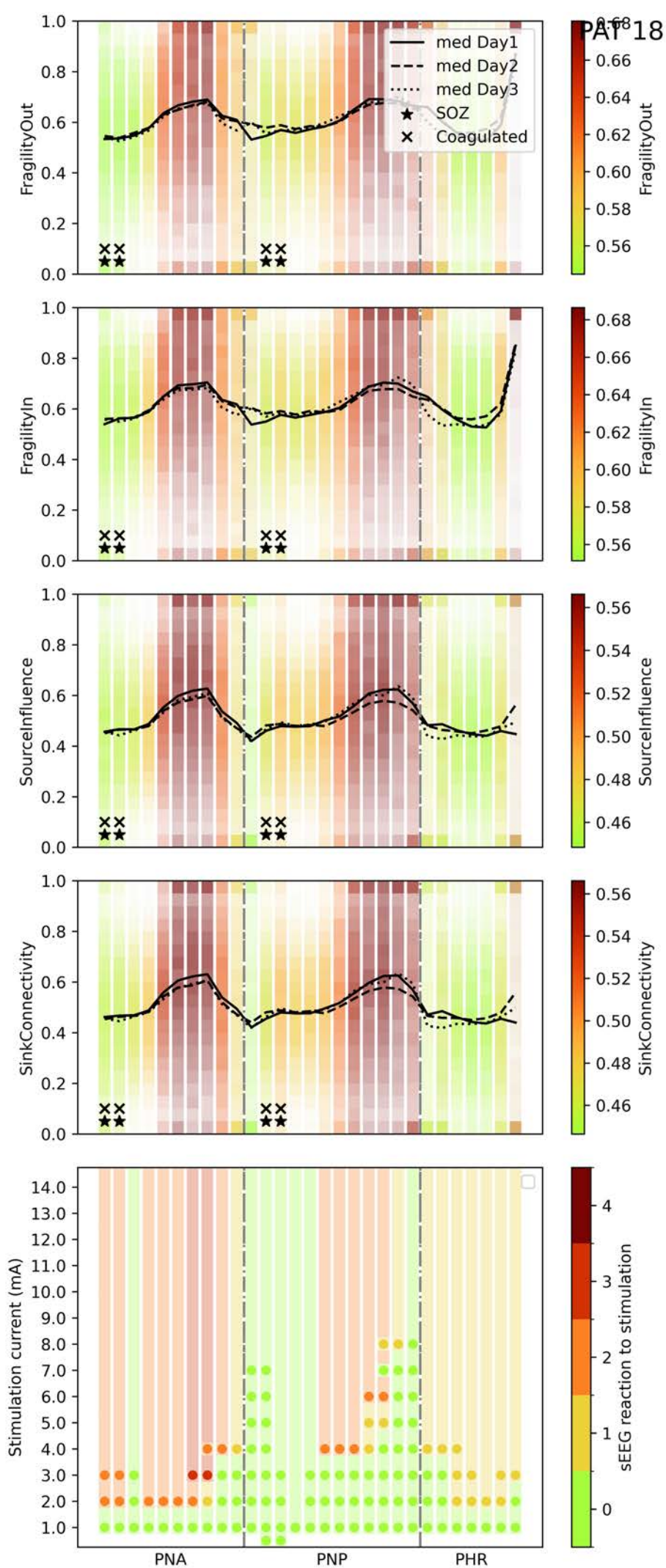

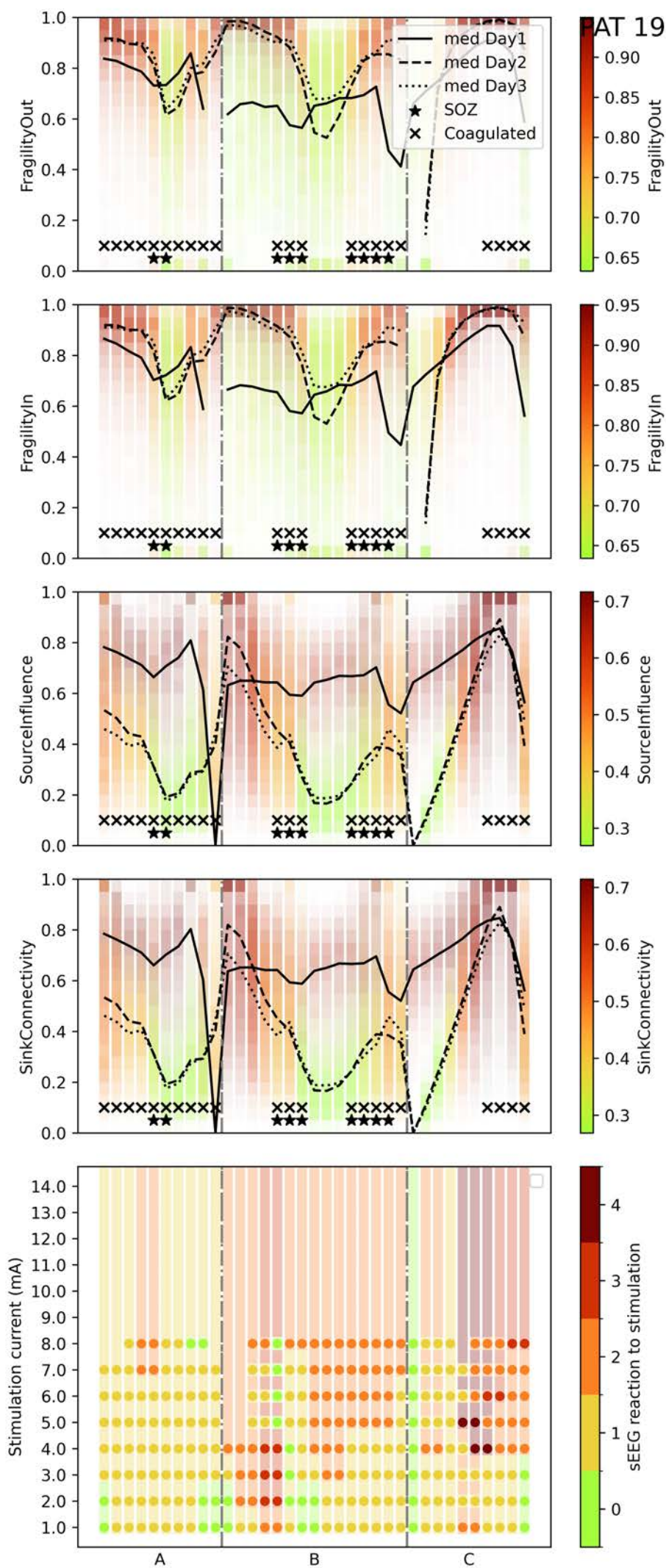

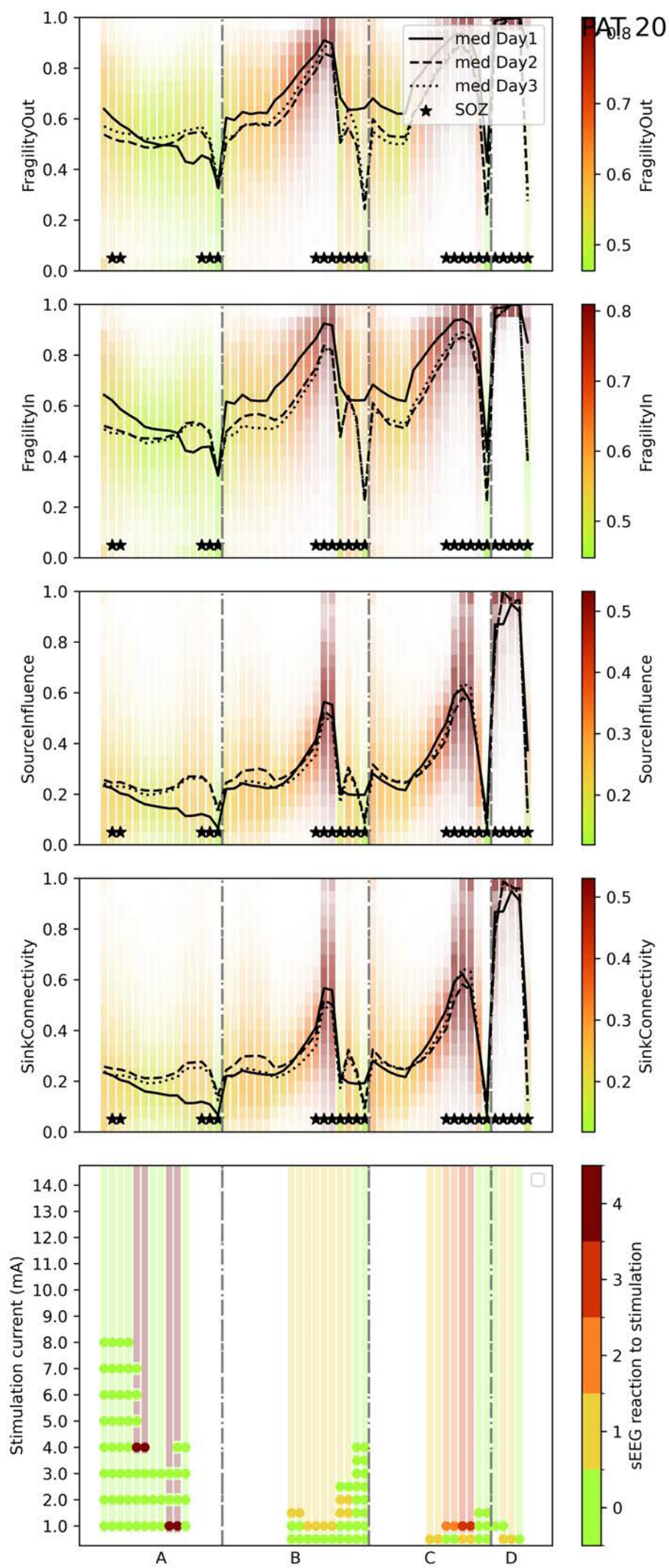

Supplement: Supplementary file 1 — Data S1: Supporting Information. [file EPI4-9999-0-s001.zip › epi470311-sup-0004-Supinfo4.pdf]
